# Multi-sample/multi-nucleus parallel polarization and monitoring enabled by a fluid path technology compatible cryogenic probe for dissolution dynamic nuclear polarization

**DOI:** 10.1038/s41598-023-34958-3

**Published:** 2023-05-17

**Authors:** Thanh Phong Lê, Jean-Noël Hyacinthe, Andrea Capozzi

**Affiliations:** 1 LIFMET, Institute of Physics, École Polytechnique Fédérale de Lausanne (EPFL), Station 6, 1015 Lausanne, Switzerland; 2 Image Guided Intervention Laboratory, Department of Radiology and Medical Informatics, University of Geneva, 4 Rue Gabrielle – Perret – Gentil, 1211 Geneva, Switzerland; 3 Geneva School of Health Sciences, HES-SO University of Applied Sciences and Arts Western Switzerland, 47 Avenue de Champel, 1206 Geneva, Switzerland; 4 HYPERMAG, Department of Health Technology, Technical University of Denmark, Building 349, 2800 Kgs Lyngby, Denmark

**Keywords:** Applied physics, Techniques and instrumentation, Cancer imaging, Cancer metabolism

## Abstract

Low throughput is one of dissolution Dynamic Nuclear Polarization (dDNP) main shortcomings. Especially for clinical and preclinical applications, where direct ^13^C nuclei polarization is usually pursued, it takes hours to generate one single hyperpolarized (HP) sample. Being able to hyperpolarize more samples at once represents a clear advantage and can expand the range and complexity of the applications. In this work, we present the design and performance of a highly versatile and customizable dDNP cryogenic probe, herein adapted to a 5 T “wet” preclinical polarizer, that can accommodate up to three samples at once and, most importantly, it is capable of monitoring the solid-state spin dynamics of each sample separately, regardless of the kind of radical used and the nuclear species of interest. Within 30 min, the system was able to dispense three HP solutions with high repeatability across the channels (30.0 ± 1.2% carbon polarization for [1-^13^C]pyruvic acid doped with trityl radical). Moreover, we tested multi-nucleus NMR capability by polarizing and monitoring simultaneously ^13^C, ^1^H and ^129^Xe. Finally, we implemented [1-^13^C]lactate/[1-^13^C]pyruvate polarization and back-to-back dissolution and injection in a healthy mouse model to perform multiple-substrate HP Magnetic Resonance Spectroscopy (MRS) at 14.1 T.

## Introduction

Hyperpolarization via dissolution Dynamic Nuclear Polarization (dDNP) ^1^ was invented in 2003 by Ardenkjaer-Larsen and co-workers to enhance, by several orders of magnitude, the liquid-state Nuclear Magnetic Resonance (NMR) signal of biomolecules relevant to investigate fundamental physiological processes as aberrant glycolytic metabolism in cancer tissues, fatty acids cardiac metabolism, and organs perfusion ^2–4^. Although all NMR active nuclear spins can be hyperpolarized via dDNP ^5–9^, during the last two decades, the technique has proved itself as the most effective and versatile key of access to ^13^C MR Imaging and Spectroscopy with second short time resolution ^3^. The latter unleashed a new range of applications aimed at investigating several enzymatic pathways and other physiological properties in real time for preclinical^2,10,11^ and clinical studies ^12–15^. Unfortunately, this unprecedented MR signal enhancement does not come without a price. Low throughput is one of dDNP main shortcomings. Indeed, prior to dissolution of the sample from the polarizer or a transportation device ^1,16–18^, the polarization transfer from the electron spins, added to the sample in form of stable or labile radicals ^19–25^, to the nuclear spins happens at low temperature (i.e. 0.8–1.5 K) ^1,26,27^ and moderate magnetic field (i.e. 3–10 T) ^28–30^, by means of microwave irradiation close to the radical’s Electron Spin Resonance (ESR) ^31^. These temperature/field conditions, needed to reach close to unity electron spin polarization, extend the build-up time of X-nuclei polarization to the range of hours.

Concurrently, applications as the study of the pharmacodynamics of an anti-cancer tracer ^32^, examination of the heart metabolism after infarction ^33^, multiple compounds injection for the combined investigation of diverse physiological processes ^34–36^, are few examples that greatly benefit from the possibility to produce, inject and monitor the fate of different hyperpolarized solutions within minutes.

Cross-polarization schemes from hyperpolarized protons ^37^ can decrease the polarization time of X-nuclei to tens of minutes, but the high B_1_ requirements limit the efficiency of this approach to small samples, and the turnover of the latter remains time consuming. Therefore, across the years, dDNP polarizers with multi-sample capability have been developed ^38,39^, in particular employing the Fluid Path (FP) technology ^26,40^, because of its versatility and reduced cryogenic heat load.

Usually, in dDNP “one size does not fit all”. Sample preparation and polarization conditions must be optimized for different substrates, nuclear species, and radicals. Hyperpolarizing more samples with different formulations is a non-trivial task, especially if different radicals are involved. When using radicals traditionally employed in dDNP (e.g. nitroxyl and trityl), thermal mixing or cross effect are the dominant polarization transfer mechanisms ^41^. In this case the appearance of the DNP spectrum is strongly dependent on the ESR properties of the radical rather than the Larmor frequency of the nuclei ^42–44^. If the radicals used have very different g-tensors, the DNP spectra for a given nucleus/radical pair can be far apart precluding the possibility to polarize simultaneously the samples.

Hence, possible advanced applications that involve the polarization of different nuclei or simply different ^13^C labelled substrates would benefit greatly from selective NMR detection.

In the present work, we detail the design and performance of a highly versatile and customizable dDNP cryogenic probe, herein adapted to a 5 T “wet” preclinical polarizer ^45^, that, employing a Custom Fluid Path (CFP) ^18,46^, can not only co-polarize up to three samples, but it is also capable of monitoring the solid-state dynamic of each of them separately, thanks to dedicated pseudo-Alderman-Grant (AG) coils and multi-nucleus parallel Nuclear Magnetic Resonance (NMR) acquisition on three distinct channels. We tested the system for the generation of three HP solutions of the same substrate (i.e. [1-^13^C]pyruvic acid) within a short time interval; for the simultaneous polarization and monitoring of ^13^C, ^1^H and ^129^Xe; and for [1-^13^C]lactate/[1-^13^C]pyruvate polarization and back-to-back dissolution and injection in a healthy mouse model to perform multi-substrate HP Magnetic Resonance Spectroscopy (MRS) at 14.1 T.

## Materials and methods

### Multi-sample DNP probe design and NMR setup

**Figure 1:**
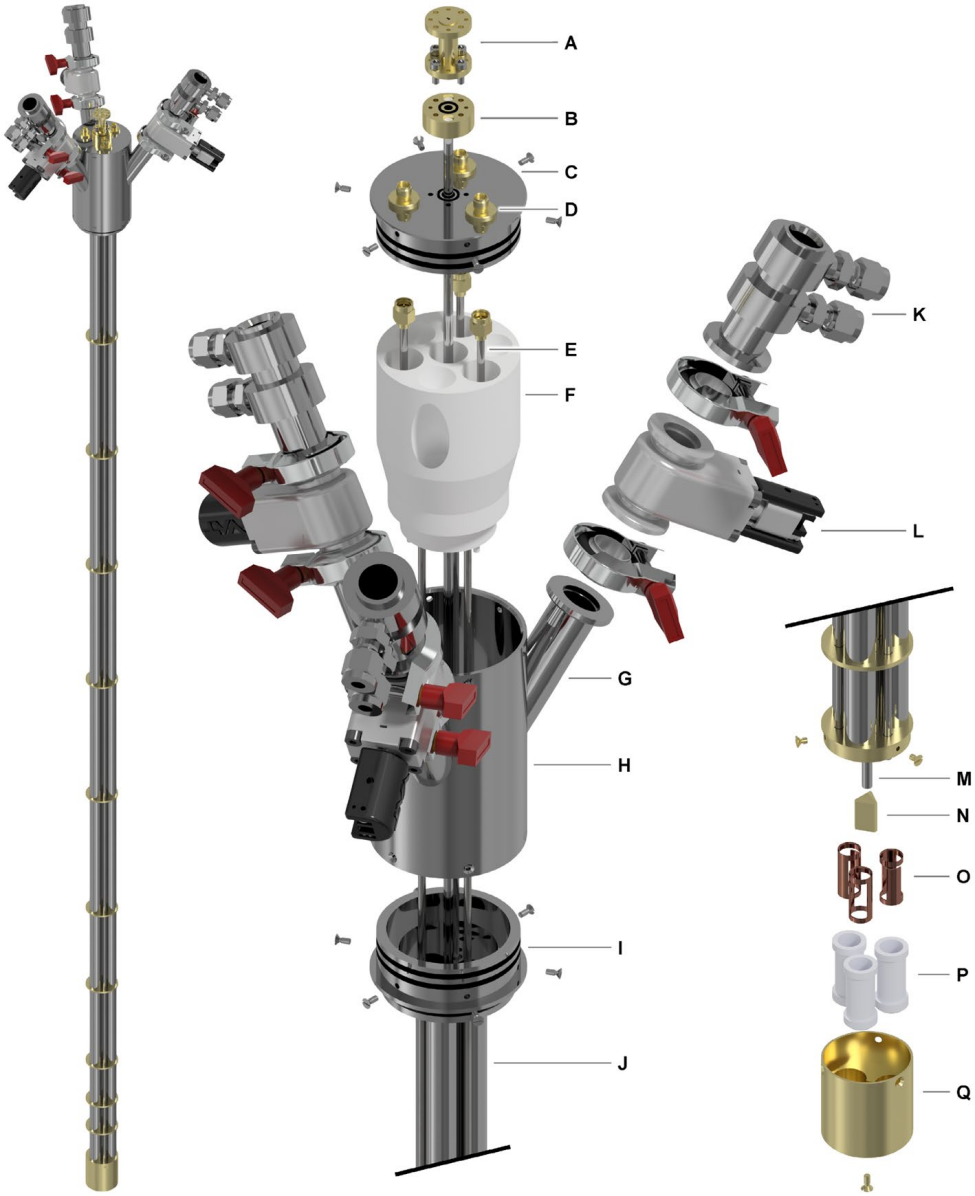
Multi-sample dDNP cryogenic probe designed to accommodate three custom fluid paths (CFPs). The main components are labelled with capital letters: WR-06 to circular 3.6 mm microwave transition (**A**); waveguide flange (**B**); top flange (**C**); SMA feedthrough connector (**D**); semi-rigid 0.141″ stainless-steel coaxial cable (**E**); 3D-printed guide (**F**); ISO-KF16 half nipple (**G**); 3-arms manifold (**H**); ISO-KF40 flange (**I**); 12.6/13.0 mm stainless-steel sample loading tube (**J**); loading chamber (**K**); ISO-KF16 gate valve (**L**); circular 3.6/4.0 mm stainless-steel waveguide (**M**); gold-plated tetrahedral microwave reflector (**N**); pseudo-Aldermann-Grant NMR coils, one for each sample (**O**); PTFE coil formers (**P**); gold-plated microwave cavity (**Q**).

In Fig. 1, we report 3D drawings of the multi-sample dDNP cryogenic probe adapted to our 5 T “wet” polarizer, whose design, functioning principle, retrofit and performance were described in detail earlier ^27,45^. All main components are indicated by capital letters.

Inspired by the SPINlab seminal paper ^26^ and the preclinical polarizer by T. Cheng et al. ^40^, sample handling is managed using the FP technology: the presence of a dynamic sealing and an air-lock compartment allows to keep the dDNP polarizer constantly at low pressure during loading, polarization and dissolution. This feature is crucial when working with more than one sample because it allows independent operations on each of them. Differently, we employ CFPs that allow more flexibility in terms of reusability and nature (i.e. liquid or solid) of the compound of interest upon loading ^9,18,45,46^. The 1470 mm long cryogenic probe is built around a 316L stainless steel 3-arms manifold (H) composed by a 58.3/68.0 mm ID/OD tube with 3 ISO-KF16 half-nipples welded on it at a 30° angle (G). The manifold is hermetically closed at the top by a stainless-steel flange (C) pressing on two 55 × 2 mm NBR O-rings. The top flange holds the microwave inlet to the waveguide (B) and three hermetic SMA connectors (D, SF-2991-6002, Amphenol SV Microwave, West Palm Beach, USA). Each ISO-KF16 half-nipple is connected to an air-lock compartment composed by an ISO-KF16 gate valve (L, Vatlock 01224-KA06, VAT, Haag, Switzerland) and a 316L stainless-steel loading chamber (K) with two hermetically glued (Araldite^®^, Huntsman, The Woodlands, USA) Swagelok connectors (SS-6M0-1-2W, Swagelok, Solon, USA) to pump on the dynamic sealing (top connector) during sample operation below the gate valve, and to flush the chamber’s volume with He gas (bottom connector) during sample operation above the gate valve. A custom-made ISO-KF40 316L stainless-steel flange (I) hermetically closes the bottom part of the manifold with two 55 × 2 mm NBR O-rings and seals the cryogenic probe to the polarizer Variable Temperature Insert (VTI, not shown). A 3D printed structure (F), placed inside the manifold, provides support and alignment for the 3.6/4.0 mm ID/OD circular 316L stainless-steel waveguide (M, Interalloy AG, Schinznach-Bad, Switzerland), the three semi-rigid coaxial cables with stainless steel outer conductor (E, 0.141SS-W-P-50, Jyebao, Taiwan) and, most importantly, guides each CFP inside one of the three 1225 mm long 316L stainless steel 12.6/13.0 mm ID/OD sample tubes (J, Interalloy AG, Schinznach-Bad, Switzerland) upon sample insertion. The tubes are welded to the bottom of the ISO-KF40 flange.

Below the ISO-KF40 flange, brazed gold-plated baffles offer mechanical support to the sample tubes, the coaxial cables and the waveguide, and reduce heat transfer via convection and radiation ^16,45^. The top of a gold-plated microwave cavity (Q) is brazed to the lower end of the sample tubes. Differently, the centrally mounted waveguide and the three coaxial cables enter the cavity and terminate, respectively, close to a microwave reflector (N) and three pseudo copper AG NMR coils (O) placed inside three PTFE coil formers (P) for electrical insulation.

A single microwave source (VCOM-06/140/1/50-DD, ELVA-1, Tallinn, Estonia) is interfaced to the waveguide with a circular 3.6 mm to rectangular WR-06 transition (A, Elmika, Vilnius, Lithuania), a 90° WR-06 E-plane bend (Elmika, Vilnius, Lithuania, not shown), and a 150 mm WR-6 straight waveguide (ELVA-1, Tallinn, Estonia, not shown).

**Figure 2:**
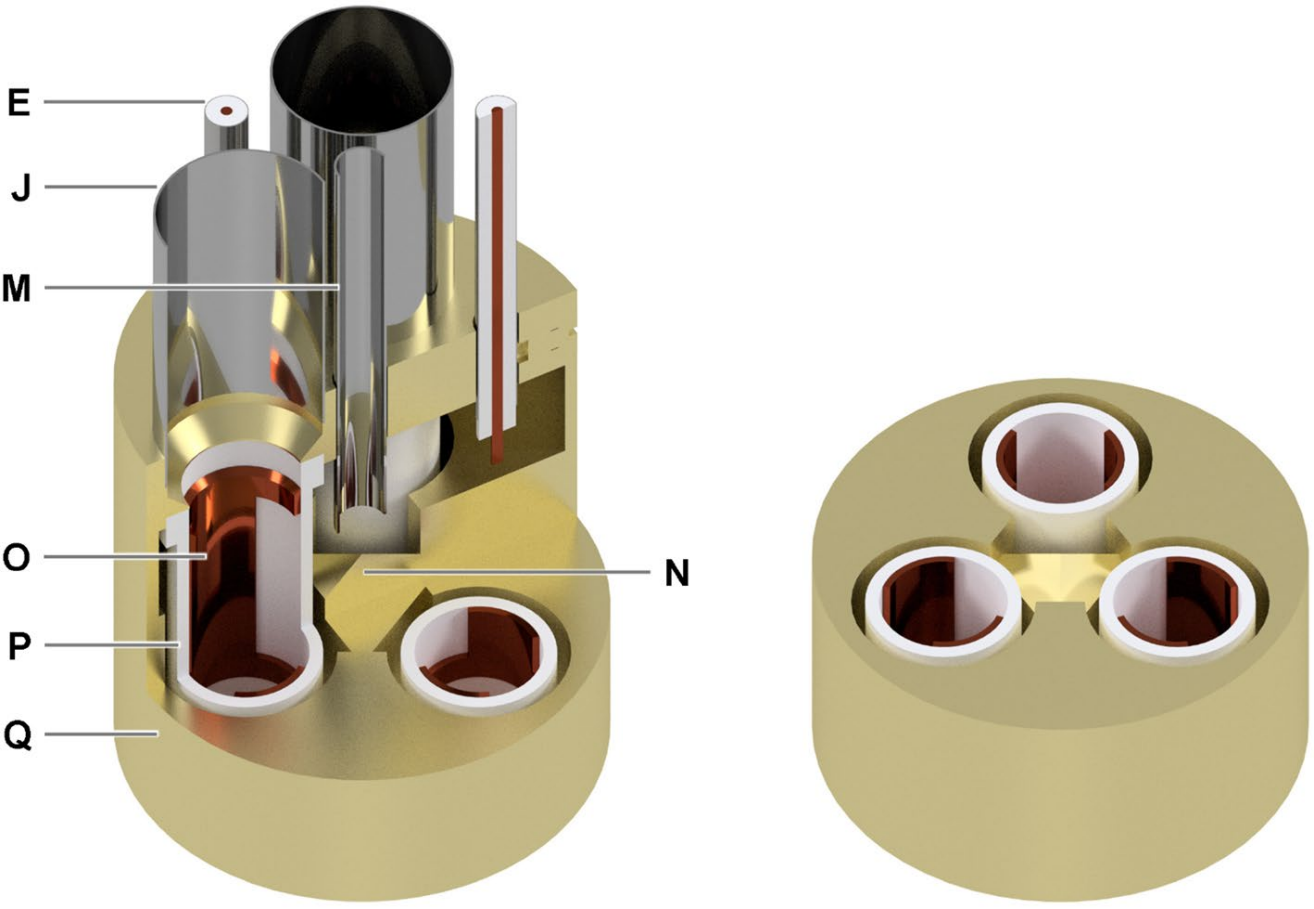
Detailed section views of the fully assembled microwave cavity. The main components are labelled with capital letters: semi-rigid 0.141″ stainless-steel coaxial cable (**E**); 12.6/13.0 mm stainless-steel sample loading tube (**J**); circular 3.6/4.0 mm stainless-steel waveguide (**M**); gold-plated tetrahedral microwave reflector (**N**); pseudo-Aldermann-Grant NMR coils, one for each sample (**O**); PTFE coil formers (**P**); gold-plated microwave cavity (**Q**).

To better understand the logic behind the microwave delivery scheme and NMR coils decoupling, in Fig. 2 we show in detail section views of the fully assembled microwave cavity. The different elements are labelled as in Fig. 1. The reflector is shaped as a tetrahedron whose top vertex is concentric to the waveguide; the bottom face is screwed onto the bottom of the cavity and the other three faces form a 45° angle with the vertical axis. In this way, the microwave beam splits in three portions that invest perpendicularly each sample through one opening of the AG coil. Each AG coil is remotely tuned and matched outside of the VTI with a coaxial cable and a tuning/matching network mounting two 1.5–250 pF piston trimmer capacitors (Knowles-Voltronics V1949, Itasca, USA) ^27,45^. The inside of the microwave cavity makes a tight fit around the coil formers. These metallic “wells” not only act as cavity reducers increasing the microwave energy density around the samples, but also shield the B_1_ of the AG coils avoiding cross talking between the three NMR channels.

The three NMR circuits are interfaced to a 3Tx/3Rx NMR console (Gecho, RS2D, Mundolsheim, France) via passive T/R switches and custom-designed low-noise (26.2 dB gain and 0.39 dB noise figure at 55 MHz) preamplifiers based on PGA-103 + (Mini-Circuits, Brooklyn NY, USA). Details about the preamplifiers design are reported in Sect. 1 of Supporting Information. RF power is provided by two RF power amplifier modules embedded inside the NMR console (5–310 MHz, 300 W, BTM00300-GammaS, Tomco, Stepney, Australia), and one external amplifier (5–200 MHz, 100 W, BT00100-Gamma, Tomco). To allow parallel NMR acquisition on the three channels for polarization build-up monitoring, microwave frequency sweep, and microwave power sweep, the existing sequences ^45^ were modified, and a new plugin for online data processing and visualization implemented (see Sect. 2 of Supporting Information). In case of homonuclear experiments three identical tuning/matching networks were used and each pair of power amplifier output/preamplifier gain was carefully adjusted to provide the same B_1_ and signal amplification across the channels. Using parallel transmission and reception NMR channels, the excitation pulses were sent on the three samples at the same time, then their respective signal was simultaneously detected. During heteronuclear experiments, specific tuning/matching networks were used to cover the needed frequency range. The three samples were measured sequentially with a short delay (< 1 ms) because of the single local oscillator (LO) architecture of the spectrometer.

### dDNP of [1-^13^C]pyruvic acid

Consistency among the three channels, in terms of polarization in the solid state and after dissolution, was investigated using [1-^13^C]pyruvic acid (Sigma Aldrich, Buchs, Switzerland) doped with 15 mM of OX063 trityl radical (Albeda Research, Copenhagen, Denmark), hereafter referred as PA-sample. 5 µL of PA-sample was loaded into three different CFPs, together with NaOH at stoichiometric ratio (7.2 µL of 10 M NaOH in H_2_O), as earlier described ^45^. The CFPs were inserted one by one inside the polarizer.

Once the working base temperature was steadily reached, the ^13^C NMR signal (Larmor frequency of 53.43 MHz) was simultaneously measured on the three channels as a function of the microwave frequency (from 139.8 to 140.0 GHz in steps of 10 MHz). This experiment, repeated only once (n = 1), allowed us to obtain the so-called ^13^C DNP sweep from the three sample slots simultaneously. For each frequency step, the samples were exposed to microwave irradiation for 10 min. The power was kept constant at 55 mW across the microwave frequency span. At the end of each irradiation period, the NMR signal from each slot was acquired using a 30° hard pulse and then destroyed sending a comb of 1000 hard pulses of 10°, while switching off the microwaves to avoid any repolarization.

NMR signal enhancement as a function of microwave power (i.e. a microwave power sweep) was performed at microwave frequency of 139.87 GHz to investigate power density differences between the three sample slots (n = 1). The power was increased from 1 to 3 mW in steps of 1 mW and from 5 to 60 mW in steps of 5 mW. The final step was set at 63 mW, which is the maximal power output of the microwave source at this frequency. Before each new power step, the NMR signal of each sample was destroyed sending a comb of 1000 hard pulses of 10°.

From the DNP frequency sweep and microwave power profile, we could find the microwave irradiation parameters providing the best NMR signal enhancement. These two values (i.e. 139.87 GHz and 60 mW) were used to investigate differences in maximum NMR signal enhancement and NMR signal enhancement dynamic across the channels. The NMR signal dynamic was simultaneously acquired on each sample using a 5° hard pulse (n = 2). This procedure was repeated every 120 s to follow the NMR signal build-up curve. The experiment lasted at least 2.5 h, more than four times the build-up time constant (i.e. at least 98% of the polarization plateau, result of a mono-exponential curve fit).

After polarization, the three samples were dissolved back-to-back with a 15 min interval between consecutive samples. Each HP solution was automatically transferred to a 1.05 T benchtop NMR spectrometer (SpinSolve ^13^C/^129^Xe Ultra, Magritek, Aachen, Germany) to measure its liquid-state polarization and relaxation time. Each PA-sample was melted using 5.5 mL of buffer solution (40 mM TRIS, 0.3 mM EDTA in D_2_O balanced to pD = 7.6) pre-pressurized at 4 bar with He gas, heated up to 180 °C (12 bar vapour pressure) and pushed for 2.5 s with He gas at 8.0 bar over a 2.0 m long, 2.0/3.0 mm ID/OD PTFE tube into a separator-infusion pump ^27^, placed on top of the benchtop spectrometer (see Sect. 3 of Supporting Information for detail about the dissolution device). After a settling time of 0.5 s, approx. 750 µL of solution was injected into a 5 mm NMR tube already placed inside the spectrometer. The acquisition started 6 s after the beginning of the injection and was performed sending 1° pulses every 3 s for 60 times. The decay of the HP NMR signal was fitted with a mono-exponential function to measure the ^13^C T_1_ of [1-^13^C]pyruvate at 1.05 T. It is important to notice that the NMR solid-state signal on three channels was continuously acquired until the last CFP was removed from the polarizer sample space to evaluate possible cross-talking between the coils.

Upon measurement of the thermal equilibrium signal, 2 µL of 0.5 M Gd-DO3A-butrol (Gadovist^®^, Bayer, Leverkusen, Germany) was added to the solution collected inside each NMR tube to reduce the ^13^C relaxation time of pyruvate. The thermal NMR signal was averaged for 1024 times using 90° hard pulses every 3 s. The liquid-state DNP enhancement was calculated from the ratio between the HP and thermal equilibrium NMR signals and rescaled for the difference in the flip angle of the pulses (n = 2 for each slot).

The maximum solid-state polarization was back calculated from the liquid-state value using the [1-^13^C]pyruvate T_1_ measured at 1.05 T and taking into account a delay of 9 s (2.5 s push time + 0.5 s settling time in the separator-infusion pump + 1 s push time into the NMR tube + 5 s settling time in the NMR tube) between dissolution and onset of the liquid-state acquisition.

### Evaluation of cryogenic performance

To compare the cryogenic performance of the multi-sample CFP compatible dDNP cryogenic probe to its equivalent for one sample only ^45^, sample space temperature and sample space pressure were measured over time after insertion of the CFPs in two different experiments: monitoring of the above-mentioned parameters during simultaneous DNP sweeps of three samples until depletion of all liquid He inside the cryostat (n = 1); monitoring of the above-mentioned parameters during solid-state polarization of three samples and back-to-back dissolutions until no sample was left inside the cryostat (n = 1). Prior to each experiment, the polarizer VTI was cooled down and used for half a day to lower the temperature of the two cryostat’s radiation shields to 200 K (outer shield) and 100 K (inner shield). Then the VTI was filled completely with 1300 ml of liquid He, and the sample space pumped on by a 253 m^3^/h root pump (Ruvac WAU 251, Leybold, Cologne, Germany) backed by a 65 m^3^/h rotatory pump (Trivac D65B, Leybold, Cologne, Germany). Measurements started when the sample space pressure dropped below 1 mbar. The temperature sensor used was a ruthenium oxide (RuO) resistor (10 kΩ at room temperature, RX-103A, Lake Shore Cryotronics , Westerville, OH, USA). The pressure sensor used was a capacitive gauge (CTR100 230301, Oerlikon Laybold Vacuum, Köln, Germany).

### Simultaneous DNP monitoring and optimization of samples prepared with different nuclei and radicals

The ability to co-polarize and simultaneously monitor the NMR signal dynamic of samples containing different target nuclei during a DNP experiment was tested on ^13^C, ^129^Xe and ^1^H using the following preparations:

- ^*13*^*C as target nucleus*: 20 μL of 4.4 M sodium-L-[1-^13^C]lactate (Sigma Aldrich, Buchs, Switzerland) doped with 20 mM OX063 (Albeda Research, Copenhagen, Denmark), 1 mM Gd-DO3A-butrol (Gadovist^®^, Bayer, Leverkusen, Germany) and dissolved in H_2_O:glycerol 1:1 (v:v), hereafter referred as Lac-sample. This sample was also dissolved and transferred inside the benchtop NMR spectrometer to measure its liquid-state polarization (n = 3) as described in the former paragraph.
- ^*1*^*H as target nucleus*: 50 μL of UV-light irradiated 27.8 M H_2_O in glycerol-d_8_:pyruvic acid 3:2 (v:v), hereafter referred as H_2_O-sample. The detailed sample preparation was described in a former publication ^9^; all chemicals were purchased from Sigma Aldrich, Buchs, Switzerland.
- ^*129*^*Xe as target nucleus*: 200 μL of 2.85 M natural abundance Xe and 30 mM 2,2,6,6-Tetramethyl-piperidin-1-oxyl (TEMPO) dissolved in melting isobutanol, hereafter referred as Xe-sample. The detailed sample preparation was described in a former publication ^5^; all chemicals were purchased from Sigma Aldrich, Buchs, Switzerland.

Each sample was loaded inside a different CFP and inserted into the polarizer. The NMR circuits were all matched to 50 Ω and slot 1, slot 2 and slot 3 tuned to the Larmor frequency of ^13^C (53.43 MHz), ^1^H (212.48 MHz) and ^129^Xe (58.78 MHz), respectively. Microwave frequency sweeps were performed as earlier described, but the frequency span was increased to 139.80–140.15 GHz. Willing to co-polarize samples containing different radicals, the influence of microwave frequency modulation was also investigated, and three more sweeps, with 20, 40 and 60 MHz of frequency modulation peak-to-peak amplitude at 1 kHz modulation rate, were performed. Finally, the polarization build-up at optimal microwave irradiation for the three samples was monitored acquiring the NMR signal by means of a 2.5° hard pulse on each channel. Since the local oscillator of the NMR spectrometer can generate one frequency at a time upon demodulation of the received signal, a delay of 330 µs across the three channels was introduced in the NMR sequence (see Supporting Information). This scheme was repeated every 120 s to follow the growth of the NMR signal of the samples.

### Sequential in vivo cerebral HP-MRS

We tested the ability to perform co-polarization of different compounds and back-to-back dissolution/injection in a preclinical environment using [1-^13^C]pyruvate and [1-^13^C]lactate to perform multi-substrate HP MRS in a healthy mouse model.

Animal experiments were conducted according to federal and cantonal ethical guidelines and approved by the local regulatory authorities (Service de la consommation et des affaires vétérinaires, Canton de Vaud, Switzerland), licence number VD2017.6. Male C57BL/6 J mice (10 weeks, Charles River, France) were maintained in a temperature- and humidity- controlled animal facility, a 12 h light/dark cycle and free access to food and water. Moreover, all animal experiments were conducted according to Federal and local ethical guidelines and complied with the ARRIVE guidelines.

**Figure 3:**
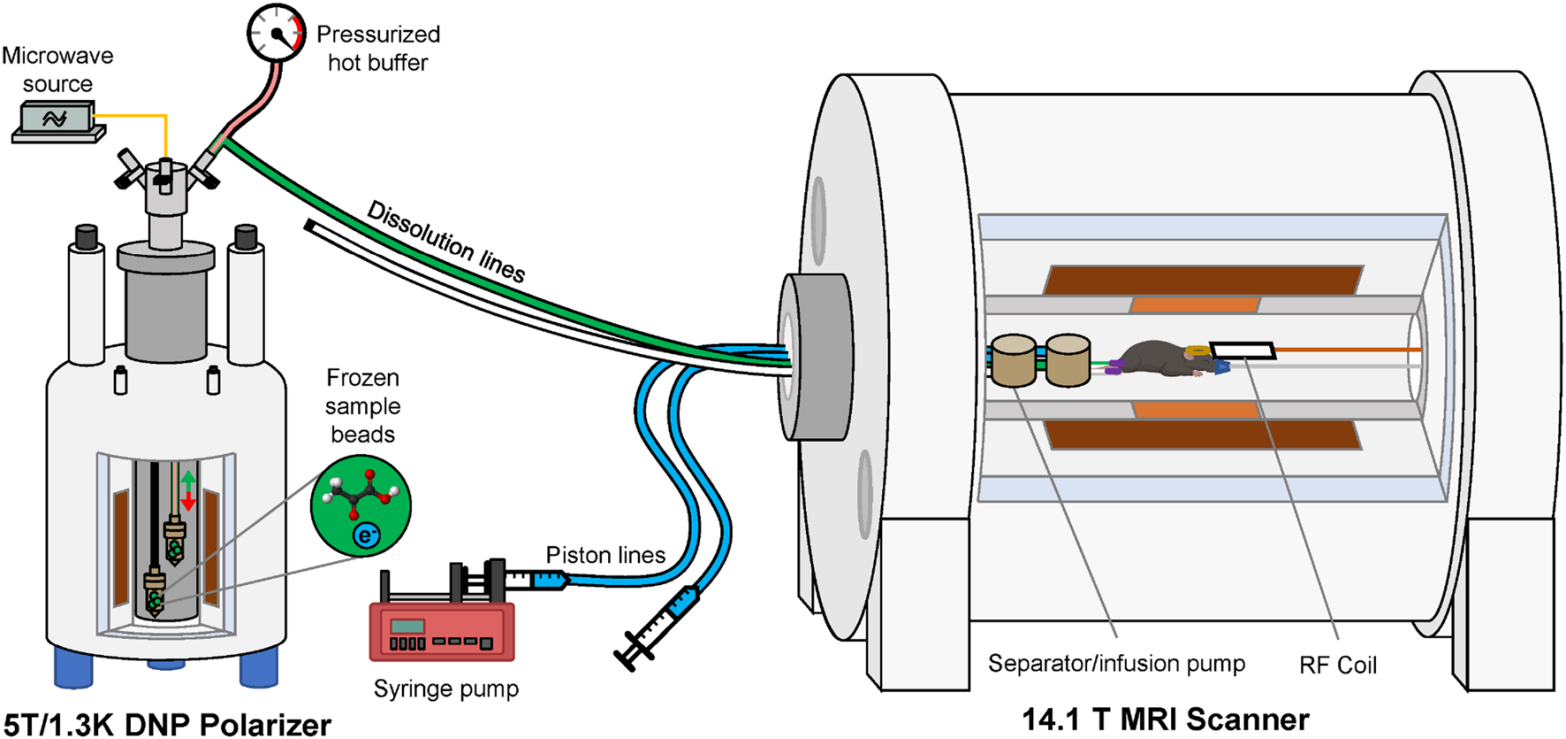
Setup for multiple injections into a healthy mouse model. The “wet” 5 T/1.3 K DNP polarizer is equipped with a multi-sample cryogenic probe loaded with two CFPs. Two separate dissolution lines transfer the HP solutions to two distinct separator-infusion pumps placed inside the MRI scanner. One syringe pump drives the injection of the HP solutions into the rat, pushing on separate water filled piston lines.

In Fig. 3 we report a sketch of the experimental setup. The multi-sample dDNP probe was loaded with two CFPs. One contained 65 μL of Lac-sample and the other 20 μL of PA-sample. Therefore, only the build-up in slot 1 and slot 2 was monitored via NMR. The samples were exposed to microwave irradiation for up to 3 h (139.86 GHz and 63 mW).

In the meantime, a healthy C57BL6/J male mouse (25.1 g) was anaesthetized with 2% isoflurane in 60% oxygen, and a catheter placed in the left femoral vein to inject the HP solutions. The mouse was immobilized with a stereotaxic device, and a ^1^H quadrature/^13^C linear (14/11 mm) surface coil was placed above the head. Two separator-infusion pumps ^27^ were simultaneously connected to the catheter using a flangeless T-junction (P-712, IDEX Health & Science, Lake Forest, IL, USA) and prefilling the 125 μL of dead volume with a phosphate-buffered saline (PBS) solution. In this way, the animal did not need to be handled between injections. Each separator-infusion pump was driven in turn using a syringe pump (NE-1010, New Era Pump Systems, Farmingdale, NY, USA) programmed to inject 450 µl through the catheter, including the dead volume. MR experiments were performed in a 14.1 T/260 mm pumped horizontal bore magnet (Magnex Scientific, Yarnton, UK) interfaced to a BioSpec Advance NEO MRI console (Bruker BioSpin, Ettlingen, Germany). Prior to ^13^C MRS, field map-based shimming was used to optimize B_0_ homogeneity in the mouse brain.

Once the whole setup was in place and the animal ready for injection, the Lac-sample was dissolved first using 5.5 mL of D_2_O pre-pressurized at 4 bar with He gas, heated up to 180 °C (12 bar vapour pressure) and pushed for 5 s with He gas at 10 bar over a 6 m long, 2.0/3.0 mm ID/OD PTFE tube to transfer the lactate HP solution to one separator-infusion pump. A 325 µl bolus of 80 mM HP [1-^13^C] lactate was intravenously injected into the mouse at a rate of 650 μL/s. Immediately, unlocalized cerebral ^13^C MRS was acquired using 30° BIR-4 pulses every 3 s during 3 min. 15 min after the first dissolution, the PA-sample was similarly dissolved in a buffer solution (60 mM TRIS, 0.3 mM EDTA in D_2_O balanced to pD = 7.6) and transferred to the second separator-infusion pump. A 325 µl bolus of 80 mM HP [1-^13^C]pyruvate was injected and measured as the previous sample.

### Statistical analysis

When experiments were repeated more than once, numerical results are reported as mean ± standard deviation.

## Results and discussion

### Cryogenic, DNP and NMR performance of the multi-sample probe

**Figure 4:**
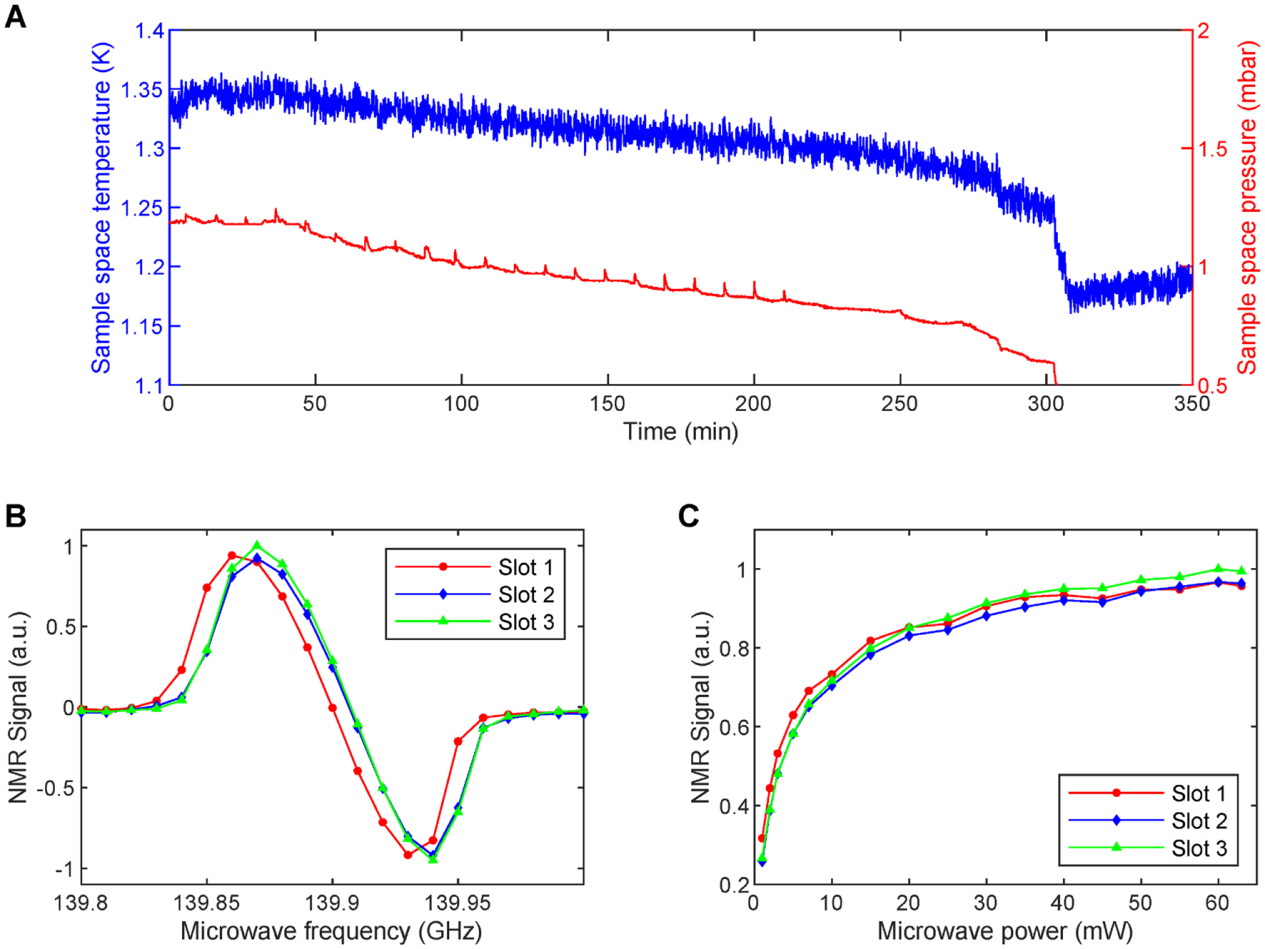
Temperature and pressure behaviour inside the polarizer sample space during the microwave frequency sweep (**A**). Microwave frequency sweeps at 5 T and 1.325 ± 0.025 K measured simultaneously on three equal volume (5 μL) PA-samples with a microwave power output of 55 mW; the NMR signal intensity of the curves was normalized according to the highest value measured across the three slots. The lines connecting the data points help guiding the eyes (**B**). Microwave power sweeps measured at 139.87 GHz for the same samples; the NMR signal intensity was normalized to the highest value of all curves. The lines connecting the data points help guiding the eyes (**C**).

**Figure 5:**
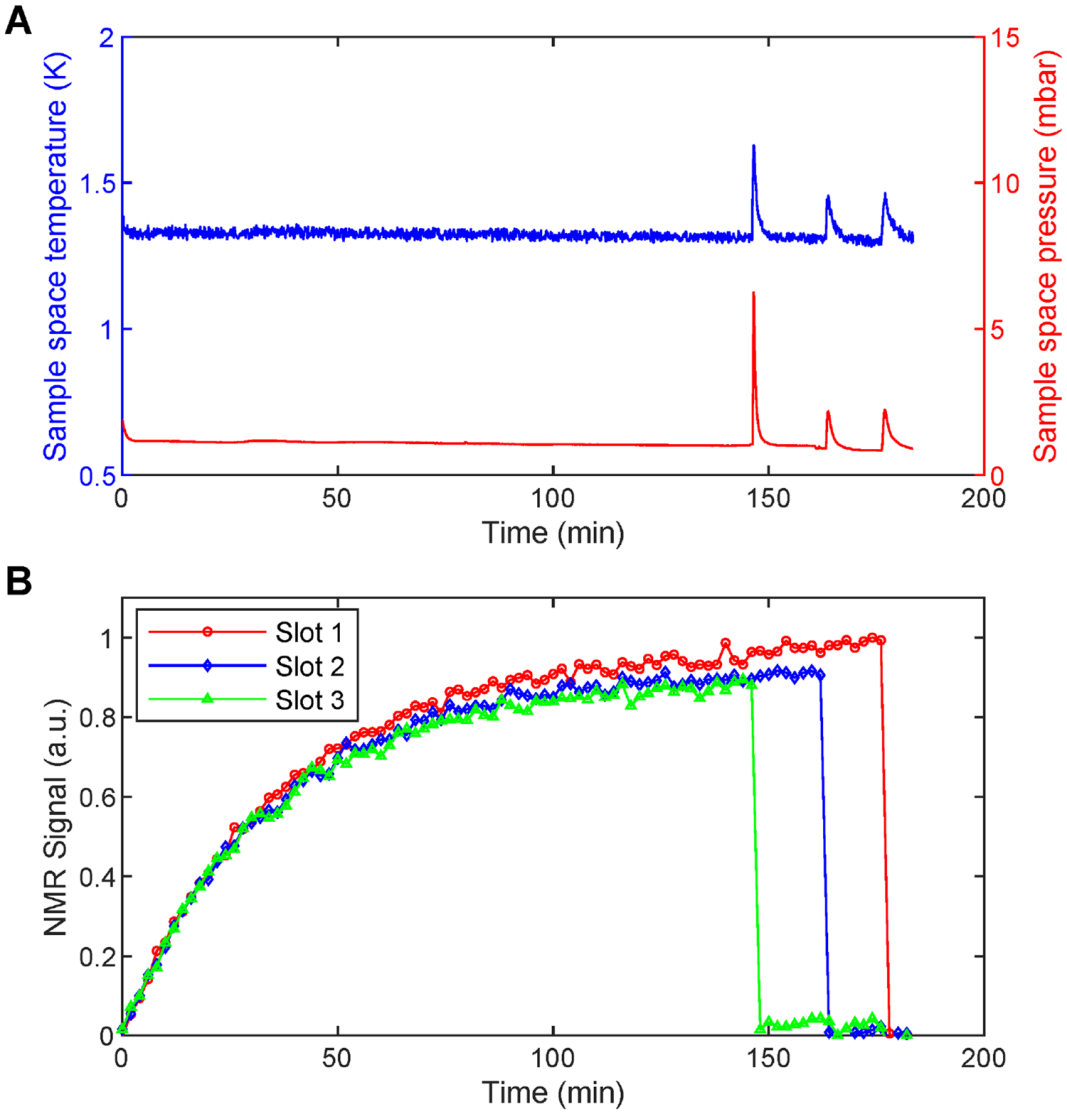
Monitoring of the cryogenic (**A**) and NMR behaviour (**B**) upon microwave irradiation at constant frequency (139.87 GHz) and constant power (63 mW) and subsequent back-to-back dissolutions. The NMR signal of the curves was normalized according to the highest value measured across the slots.

In Fig. 4 and in  Fig. 5 we summarize the performance of the multi-sample dDNP probe in terms of cryogenics, homogeneity of the microwave irradiation and Tx/Rx equivalence across the three NMR channels. For this purpose, as described in Materials and Methods, we loaded each slot with 5 μL of PA-sample. As described earlier, the dDNP system works in batch mode ^27,45^, i.e. the VTI is filled to its maximum capacity with liquid He and then the temperature is lowered by reducing the pressure of the sample space.

The maximum liquid He holding time (Fig. 4A) was measured during parallel microwave frequency sweeps (Fig. 4B). The sample space was stable between 1.35 and 1.30 K over 300 min (5 h). After this time, the sudden temperature drop and subsequent temperature increase indicated the absence of any residual cryogenic liquid inside the sample space. Correspondingly, the pressure decreased from 1.2 mbar to 0.7 mbar. During the first 210 min (duration of the microwave frequency sweep), we could appreciate pressure spikes spaced by 10 min due to the train of RF pulses used to destroy the DNP signal after each microwave frequency step. We could not observe the same pattern on the temperature graph because of higher background noise of the temperature sensor with respect to the pressure sensor.

The single slot CFP compatible dDNP probe that we recently described ^45^ allowed us to work at 1.15 K with a liquid He holding time of 6 h. For the same cooling power of the VTI, the increased heat transfer generated by the multi-sample probe is due to the extra building material rather than the three samples themselves. Indeed, as previously demonstrated ^45^, the stainless steel sample loading tube and the semi-rigid coaxial cable account for 86% of the total thermal conductivity, while the CFP for less than 1%.

Signal intensity-wise, the microwave sweeps from the three slots looked alike (in Fig. 4B, signals were normalized to the highest one, see Sect. 2 of Supporting Information for unprocessed data), but the DNP spectrum in slot 1 was shifted downfield by 10 MHz with the maximum DNP enhancement appearing at 139.86 GHz instead of 139.87 GHz. Most likely, this is due to a magnetic field difference across the slots of 71.4 ppm (i.e. 3.5 G). Indeed, upon rump-up of the magnet the cryo-shims were adjusted placing a sample aligned along the vertical axis of the polarizer. Despite this small offset, the maximum available microwave power of the source, together with the presence of a tetrahedral mirror placed in front of the bottom part of the waveguide, was enough to saturate the DNP enhancement when working at 139.87 GHz (Fig. 4C). Although compared to only 20 mW used for the single-sample dDNP probe ^45^, a threefold amount of power was required to obtain the maximum available enhancement, the power sweeps showed an even distribution of the microwaves across the slots.

This behaviour was confirmed by simultaneously monitoring the NMR signal dynamic of the three PA-samples upon irradiation at constant microwave frequency (i.e. 139.87 GHz) and power (63 mW). In Fig. 5B we show how, within 10% discrepancy, for each time point the NMR signals measured from the three slots evolved together during 150 min of microwave irradiation (see Sect. 2 of Supporting Information for unprocessed data). During this time period, the sample space temperature and pressure remained between 1.30—1.35 K and 1.0–1.2 mbar, respectively, until the first dissolution from slot 3 was performed. At this point, these two parameters increase to 1.6 K and 6.5 mbar, respectively, because of the heat load brought by the superheated buffer flowing through the CFP (Fig. 5A). The dissolutions from slot 2 and slot 1 followed delayed by 15 min each. Concerning the behaviour of the system, four aspects are noteworthy:

- After each dissolution, the cryogenic system recovered after 5 min, going back to the base temperature and pressure values (Fig. 5A).
- The temperature/pressure jump inside the sample space due to the passage of hot buffer through the CFP in one slot did not make the NMR signal to decrease in the other slots (Fig. 5B).
- The NMR signal from the slot interested by the dissolution suddenly dropped below 4% of the pre-dissolution signal intensity (see Sect. 4 of Supporting Information) confirming the absence of pronounced cross talking between the three AG coils.
- The second and third dissolutions generated a less pronounced temperature/pressure jump because of the lower liquid He level left after the first event (Fig. 5A).

### Solid-state and liquid-state polarization of pyruvic acid

**Figure 6:**
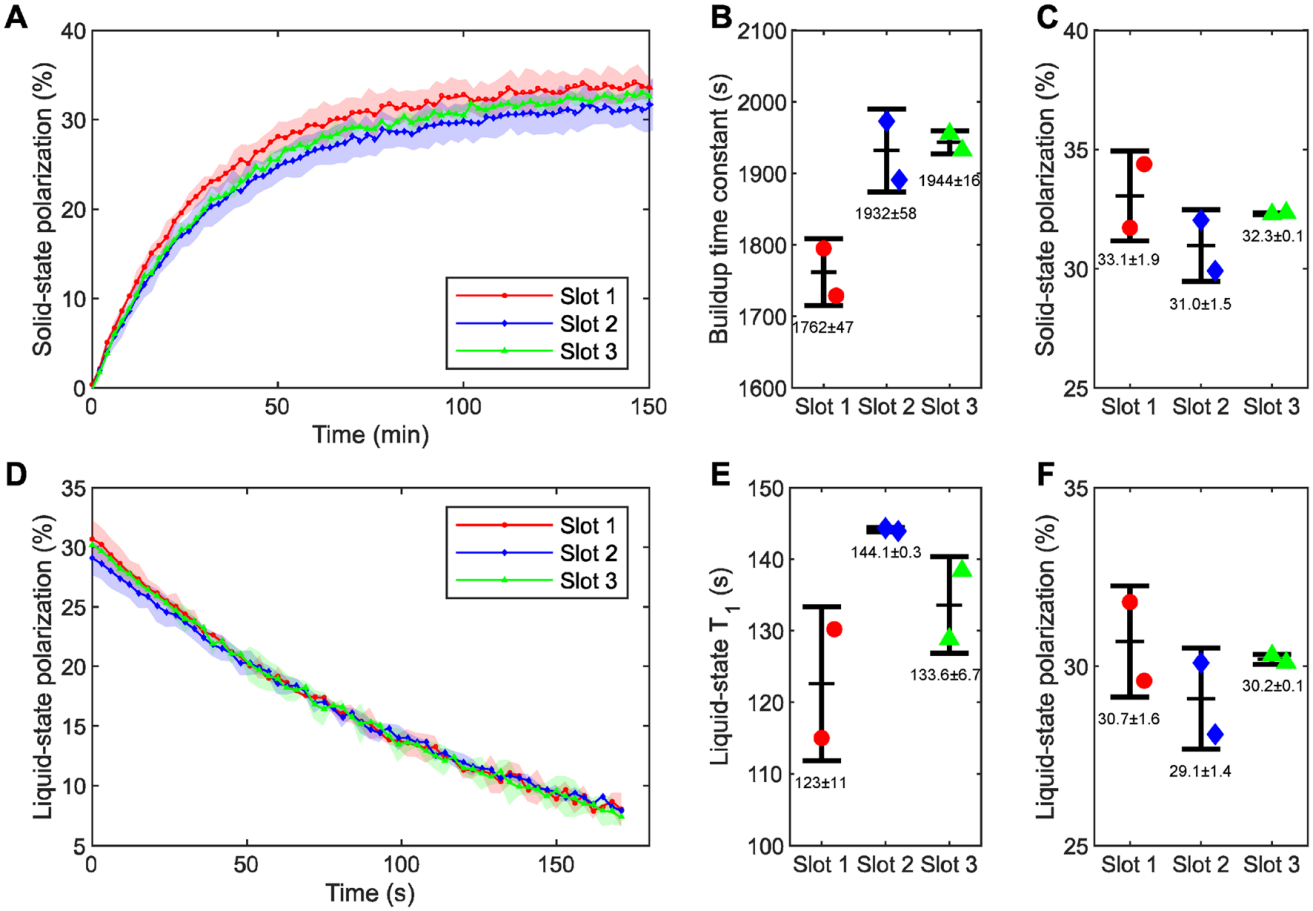
Summary of the dDNP performance of the multi-sample probe when loaded with three PA-samples (n = 2): solid-state polarization dynamics during microwave irradiation inside the polarizer (**A**); fitted mono-exponential build-up time constant (**B**); back-calculated maximum solid-state polarization from liquid-state value (**C**); liquid-state polarization dynamic after dissolution inside the benchtop NMR spectrometer (**D**); fitted mono-exponential relaxation time constant (**E**); calculated liquid-state polarization upon injection of the HP solution inside the benchtop NMR spectrometer from thermal equilibrium value. Numerical results are expressed as “mean ± standard deviation” (**F**). In panels A and D shaded areas represent the standard deviation between measurements.

In a separate set of experiments (n = 2) we measured the solid-state and liquid-state polarization obtained on the same kind of samples (i.e. three slots loaded with a PA-sample). We summarize our findings in Fig. 6. After 100 min, all samples reached at least 95% of the maximum achievable signal (Fig. 6A) with a polarization time constant of 1762 ± 47 s, 1932 ± 58 s, 1944 ± 16 s for slot 1, slot 2 and slot 3, respectively (Fig. 6B). While the build-up time constant in slots 2 and 3 was, within experimental errors, the same, the one from slot 1 was approximately 200 s shorter. Being the last point of the microwave power sweep very similar for all slots, we ascribe this discrepancy to the irradiation frequency being 10 MHz lower than the optimal value for slot 1. Indeed, it was earlier demonstrated that build-up time constants decrease moving the microwave frequency to the left/right of the positive/negative maximum of the DNP spectrum ^30^. Most importantly, the build-up was considerably shorter compared to what we measured earlier using the single-sample dDNP probe ^45^. In that case, [1-^13^C]pyruvic acid doped with 15 mM trityl showed a build-up time constant of 2745 ± 63 s. The latter is justified by the lower base temperature (1.15 K vs. 1.35 K) that could be reached using the single-sample DNP probe. Indeed, as shown in the work from Filibian et al. ^47^, the build-up time constants (${T}_{b}$) scale nicely with the relation $1/{T}_{b}={T}^{2}$, where $T$ is the temperature of the He bath. After dissolution and relaxation inside the benchtop spectrometer (Fig. 6D), we calculated an average liquid-state polarization of 30.0 ± 1.2% (Fig. 6F). With a relative error as small as 4% over six dissolutions, the system demonstrates high repeatability and equal dDNP performance across the channels. Again, compared to the single-slot probe, the slightly lower maximum achievable polarization can be justified by the difference in base temperature ^45^. The ^13^C solid-state polarization value of 32.1 ± 1.4% (Fig. 6C) was back calculated assuming a relaxation time during transfer of 130 s (Fig. 6E).

### Solid-state polarization and monitoring of different nuclei

**Figure 7:**
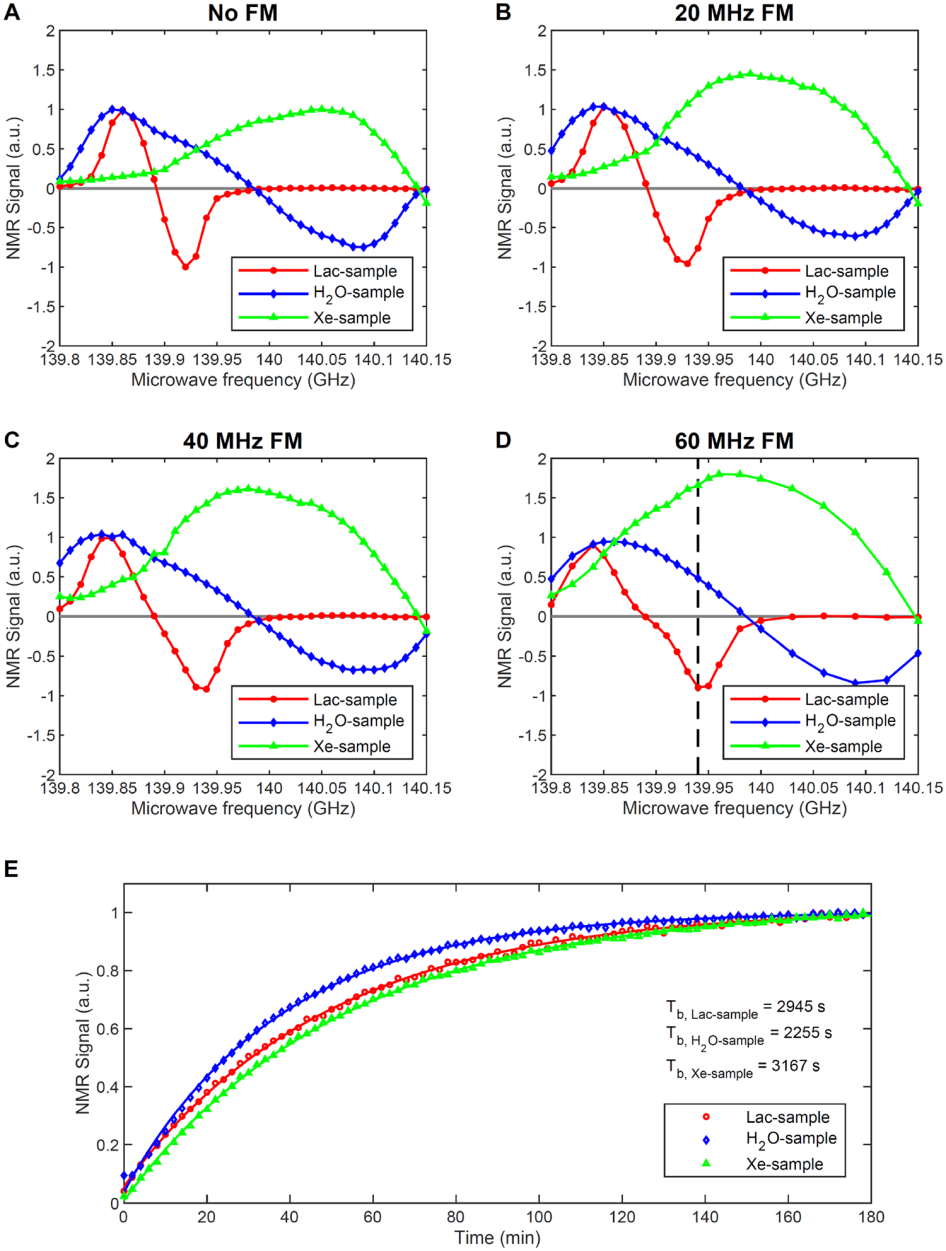
Lac-sample, H_2_O-sample and Xe-sample simultaneous DNP microwave frequency sweep without (**A**) and with 20 MHz (**B**), 40 MHz (**C**) and 60 MHz (**D**) of frequency modulation amplitude. Lac-sample, H_2_O-sample and Xe-sample simultaneous polarization build-up monitoring shining microwaves at 139.94 GHz with 60 MHz frequency modulation amplitude and 63 mW output power (**E**). The dashed line in (**D**) indicates the microwave irradiation frequency used in (**E**). The intensity of each sweep in (**A**) was normalized to 1. The signal intensity of the sweeps in (**B–D**) was normalized with respect to (**A**).

We deliberately chose an extreme case where we tried to co-polarize and monitor the Lac-sample ([1-^13^C]lactate + trityl), the Xe-sample (^129^Xe + TEMPO) and the H_2_O-sample (^1^H + lactyl radical). Figure 7A shows how monochromatic (i.e. no frequency modulation) microwave sweeps of the three samples have poor overlap, with the maximum of the Xe-sample appearing at 140.05 GHz and the maxima for the other two samples around 139.85, where the Xe-sample DNP is very far from optimal. Instead of programming the microwave source to swiftly change the output frequency, as it happens for instance in ELDOR experiments ^48^, we decided to explore the effect of microwave frequency modulation. The latter increases the breadth of the DNP spectrum, and the larger the modulation amplitude, the further apart the positive and negative DNP maxima move ^24,49^. In Fig. 7B, C and D we report the effect of increasing modulation amplitude on the DNP spectra. At 139.94 GHz, when driving the frequency output with a sine wave oscillating by 60 MHz peak-to-peak amplitude, not only the Xe-sample polarization increased by a factor of 2, but also, its positive peak became almost coincident with the negative one of the Lac-sample. At the same time, the H_2_O-sample could still be polarized at 50% of its maximum value. Therefore, we used this microwave irradiation setting to simultaneously monitor the build-up of all samples (Fig. 7E). Shining microwaves for 3 h, they all reached the maximum available polarization.

### Sequential in vivo cerebral HP MRS

In HP-MR, [1-^13^C]pyruvate is widely recognized as the golden standard to assess real-time metabolism in vivo through its conversion to lactate, bicarbonate and alanine ^4^. Nevertheless, lactate is another promising molecule, despite the least straightforward sample formulation ^50^. Differently from pyruvate, it can be injected at physiological concentration with known neuroprotective effects ^51^, it easily crosses the blood–brain barrier ^52^, and allows detecting the secondary conversions to alanine and bicarbonate through pyruvate providing a better understanding of the lactate dehydrogenase activity ^50,53^.

Therefore, to test the capability of our system to produce injectable HP solutions containing physiologically interesting metabolites with a rate much shorter compared to the usual ^13^C DNP build-up time, we chose to perform two [1-^13^C]lactate/[1-^13^C]pyruvate back-to-back injections into a healthy mouse model.

**Figure 8:**
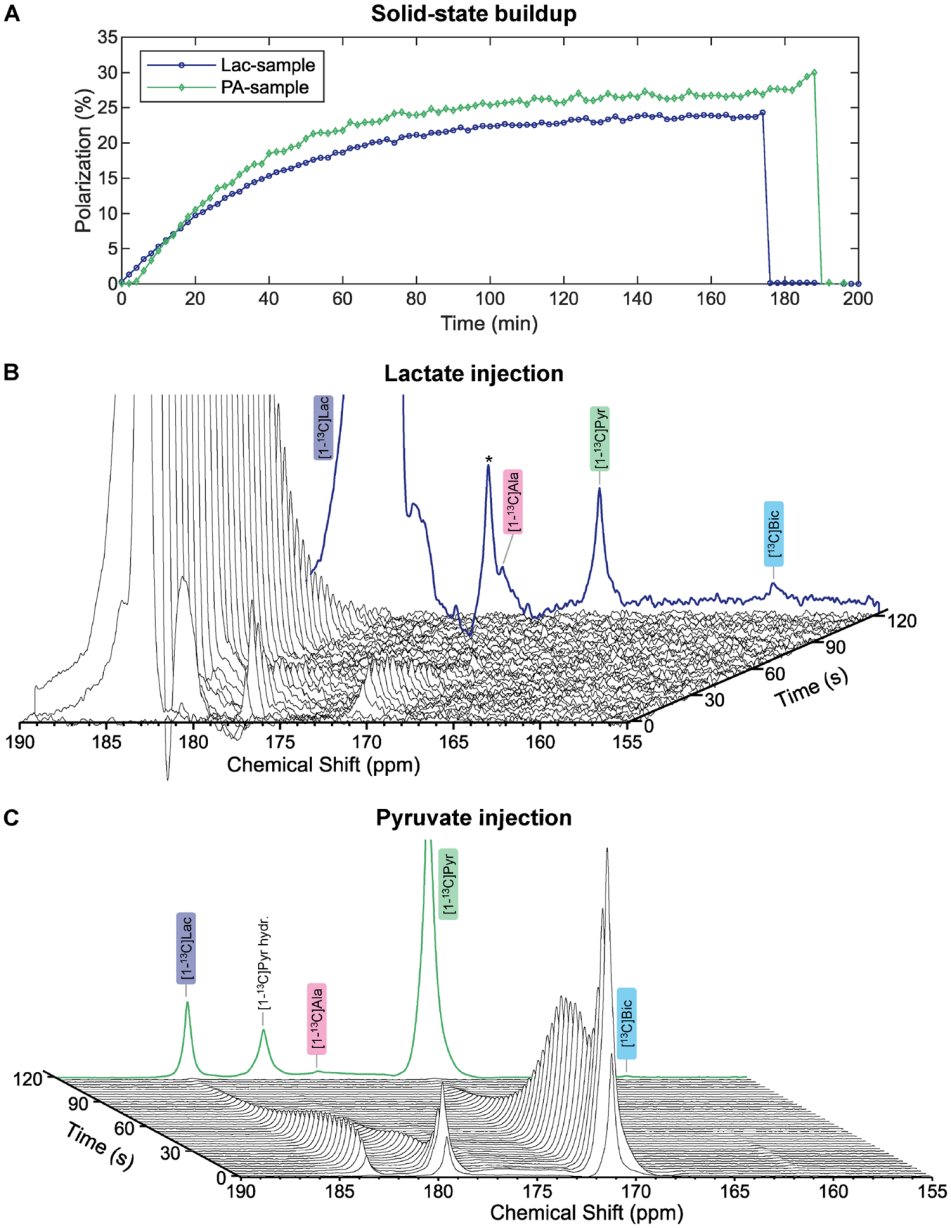
Solid-state NMR signal of the PA-sample (green) and Lac-sample (blue) simultaneously hyperpolarized and dissolved within 15 min from each other; the signal intensity was scaled to the liquid-state polarization measured in separate experiments (**A**). Unlocalized cerebral ^13^C-MRS to investigate the metabolism of HP lactate, which metabolizes into pyruvate, alanine and bicarbonate; the bold blue line represents the sum of the time course signals in the first 120 s post-injection; the peak marked with a (*) is an impurity of the stock lactate solution (**B**). Unlocalized cerebral ^13^C-MRS to investigate the metabolism of HP pyruvate, which metabolizes into lactate, alanine and bicarbonate; the bold green line represents the sum of the time course signals in the first 120 s post-injection (**C**). In (**B**) and (**C**) a line broadening of 20 Hz was applied to better display the data.

To have the same ^13^C nuclei final concentration after dissolution, the Lac-sample volume was 3.25 times larger than the PA-sample. Despite the slightly different DNP spectrum, both samples showed optimal DNP at 139.86 GHz (see Sect. 5 of Supporting Information). The slightly higher radical content compensated for the slower spin diffusion due to lower ^13^C concentration ^54^, and the Lac-sample built-up polarization approximately at the same rate as the PA-sample (Fig. 8A). In a separate set of experiments, the [1-^13^C]lactate liquid-state polarization measured inside the benchtop spectrometer was 24.3 ± 1.5% (n = 3).

The Lac-sample was dissolved first and infused into the mouse to monitor its cerebral metabolism into pyruvate, alanine and bicarbonate (Fig. 8B). 15 min later, the pyruvate sample was similarly dissolved and its cerebral conversion into lactate, alanine and bicarbonate was successfully monitored (Fig. 8C). The maximal SNR in single spectra, without applying any line broadening, was 402 and 313 for pyruvate and lactate respectively, which is consistent with the difference in liquid-state polarization level of the two samples. Nevertheless, the SNR of downstream metabolites were substantially smaller after injecting lactate compared to pyruvate. The latter was due to lower label exchange ^52^ between HP lactate to endogenous pyruvate with respect to HP pyruvate to endogenous lactate because of the difference in pool size of the two metabolites ^50,55^. The difference in endogenous pool size of the two metabolites is also at the origin of the peculiar dynamic behaviour of the pyruvate/pyruvate hydrate peak. The latter, during the first 30 s, increases, decreases and increases again because of the perturbation of the mouse respiratory and cardiac functions upon injection of the supraphysiological dose of pyruvate ^50,51^.

## Conclusions and perspectives

Regardless of their formulation, being able to optimize and/or monitor the DNP performance of more samples at once represents a powerful tool to improve and expand the scope of applications that entail multi-HP compound injections. In this paper, we have detailed the design and performance of a versatile multi-sample dDNP probe with the unique characteristic of selective NMR capability across three sample slots, and we proved its employment in a preclinical study.

Thanks to the Fluid Path technology, we were able to produce three pyruvate HP solutions within 30 min from the first dissolution, and the delay was mainly caused by the time needed for the buffer to heat-up rather than for the cryogenic system to recover. Indeed, after each dissolution, the system was able to go back to its base working temperature within 5 min. Therefore, injections with a shorter delay could be obtained by heating two or three dissolution buffers in parallel.

The working base temperature was 15% higher compared to a similarly built single-sample probe. The latter decreased the maximum achievable polarization of pyruvate accordingly. If for a wet system the increased thermal conductivity was, after all, negligible, this aspect will deserve extra care in case of implementation of the dDNP into a cryogen-free system ^40^. In that case, providing a better thermal anchoring of the probe to the radiation shields of the polarizer will for sure improve the base temperature, as well as cutting specific patterns along the stainless-steel sample tubes to reduce thermal conductivity.

Finally, we showed how microwave frequency modulation can be an easy to implement and represents a versatile tool to find the microwave irradiation “sweet spot”, when trying to simultaneously polarize samples with very different formulations.

## Supplementary Information

Supplementary Information.

## Data Availability

The authors declare that all data supporting the findings of this study are available within the paper and its Supporting Information files. Raw data are available from the corresponding author (andrea.capozzi@epfl.ch) on reasonable request. Technical drawings of the cryogenic probes are available for download from Zenodo repository at the following address (10.5281/zenodo.7937983).
