# Supplementary material for: Multi-sample/multi-nucleus parallel polarization and monitoring enabled by a fluid path technology compatible cryogenic probe for dissolution dynamic nuclear polarization
Source: Sci Rep. 2023 May 17;13:7962. doi: 10.1038/s41598-023-34958-3 (PMC10192315; doi:10.1038/s41598-023-34958-3)
Supplement: Supplementary file 1 — Supplementary Information. [file 41598_2023_34958_MOESM1_ESM.docx]

Supporting Information

**Multi-sample/multi-nucleus parallel polarization and monitoring enabled by a Fluid Path technology compatible cryogenic probe for dissolution Dynamic Nuclear Polarization.**

Thanh Phong Lê ^1^, Jean-Noël Hyacinthe ^1,2,3^, Andrea Capozzi ^1,4*^

*^1^LIFMET, Institute of Physics, École polytechnique fédérale de Lausanne (EPFL), Station 6, 1015 Lausanne (Switzerland).*

*^2^Image Guided Intervention Laboratory, Department of Radiology and Medical Informatics, University of Geneva, 4 Rue Gabrielle – Perret – Gentil, 1211 Geneva (Switzerland).*

*^3^Geneva School of Health Sciences, HES-SO University of Applied Sciences and Arts Western Switzerland, 47 Avenue de Champel, 1206 Geneva, (Switzerland).*

*^4^HYPERMAG, Department of Health Technology, Technical University of Denmark, Building 349, 2800 Kgs Lyngby (Denmark).*

**Corresponding author**

*Dr. Andrea Capozzi

EPFL SB IPHYS LIFMET

CH F0 632 (Bâtiment CH)

Station 6

CH-1015 Lausanne

T: +41 21 693 05 88

Email: andrea.capozzi@epfl.ch

ORCID: 0000-0002-2306-9049

**1. Low-noise preamplifier design**

Low noise preamplifiers were designed for performing solid-state NMR measurements. They use the E-PHEMT PGA-103+ (Mini-Circuits, Brooklyn, NY, USA) wideband amplifier, which provides a low noise figure in the relevant frequency range (50-250 MHz), sufficient gain (>22 dB), high power handling (+21 dBm), and is well documented in the amateur radio community.

A simplified schematic of the preamplifier is presented in Figure S1. Varistors RV1 and RV2 protect the amplifier U1 against electrostatic discharge. Additionally, the Schottky cross-diodes D1 protect U1 against excessive input power. The low-dropout linear regulator U2 supplies the amplifier with a constant DC voltage and requires at least 6.5 V and preferably 7.0 V input voltage to achieve optimal performances, therefore the supply voltage at J3 is preferably 7.9 V after taking the forward voltage of D3 into account. The current required is typically 120 mA in normal operation.

**Figure S1.** Simplified schematics of the preamplifier based on the PGA-103+ wideband amplifier.

Printed circuit boards (PCB) were designed in KiCad 5.2, then manufactured by a third party on double-layer FR4 boards. All components were hand-soldered on the boards (Figure S2). The board is then mounted onto an aluminum enclosure (Hammond/Eddystone 11451PSLA) holding a 1000 pF feedthrough capacitor and a ground post connected to a power supply.


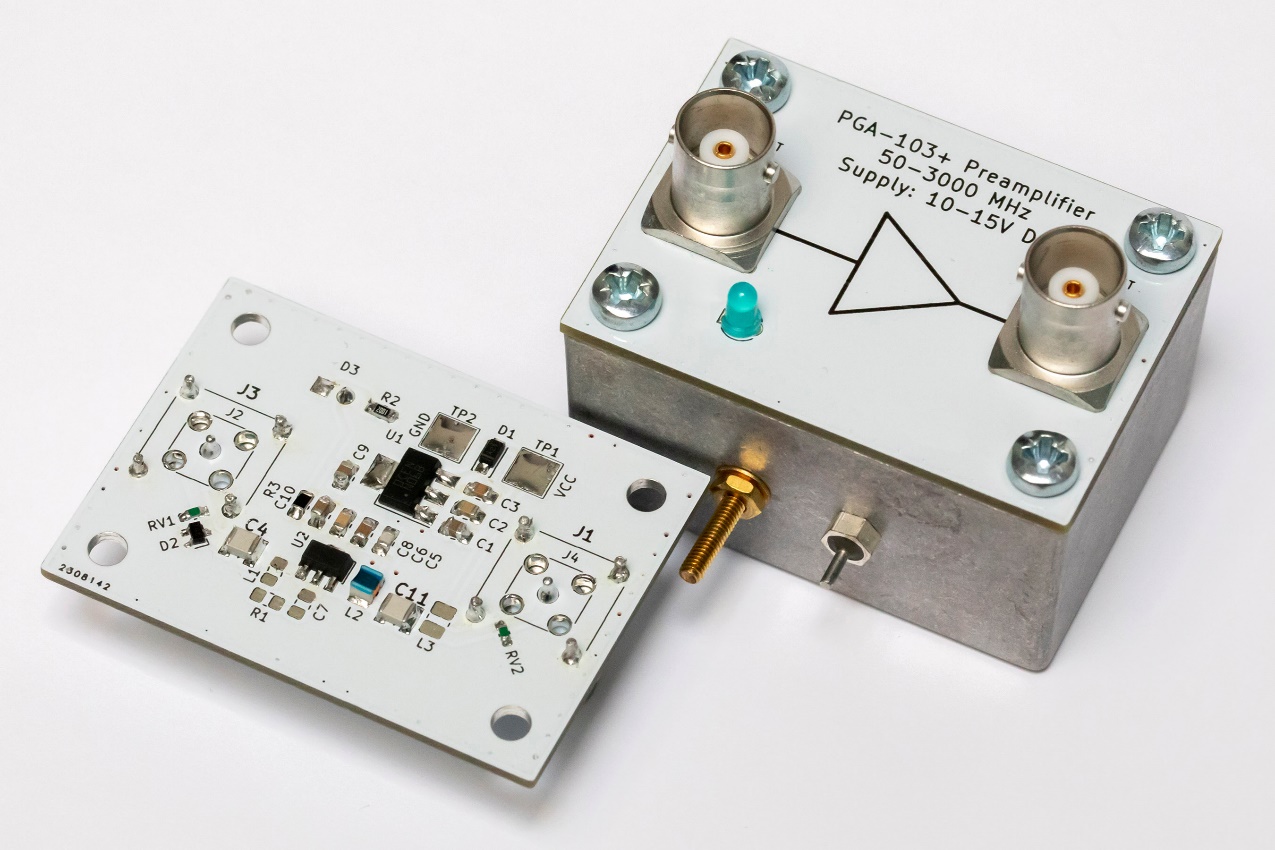


**Figure S2.** Picture of the assembled preamplifier. The PCB is mounted as the lid of an aluminum enclosure. Note that the reference designators on the assembled boards do not match the simplified schematics.

One assembled board was characterized between 10 MHz and 300 MHz using a N8973A Noise Figure Analyzer (Agilent, Santa Clara, CA, USA) and a NS346A Calibrated Noise Source (Micronetics, Hudson, NH, USA). The gain and noise figure are reported in Figure S3. At 55 MHz, approximately the ^13^C or ^129^Xe frequency at 5 T, 26.2 dB gain and 0.422 dB noise figure were measured. At 210 MHz (^1^H frequency), slightly worse performances were measured, with 23.6 dB gain and 0.646 dB noise figure.


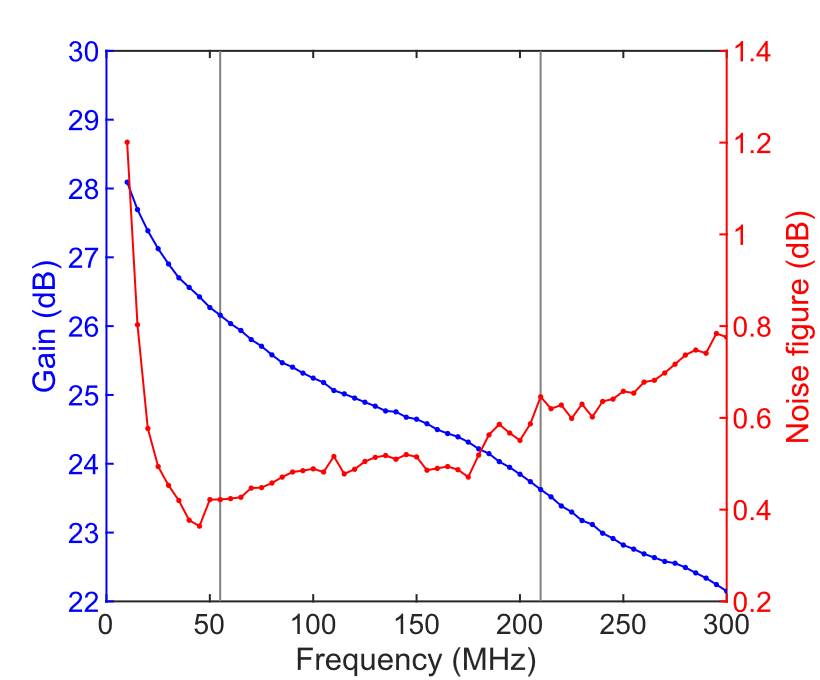


**Figure S3.** Gain and noise figure of the in-house made low-noise preamplifier

**2. NMR sequences and plugin for solid-state experiments**

In this paragraph we show the NMR sequence design schemes used to monitor simultaneously the solid-state polarization buildup of three samples with the same target nucleus (homonuclear buildup experiment, Figure S4); to monitor simultaneously the solid-state polarization buildup of three samples with different target nuclei (heteronuclear buildup experiment, Figure S5); to measure simultaneously the microwave frequency sweep for three samples with the same target nucleus (homonuclear frequency sweep experiment, Figure S7); to measure simultaneously the microwave frequency sweep for three samples with distinct target nuclei (heteronuclear frequency sweep experiment, Figure S8). Two plugins were also implemented for online signal processing and visualization of a simultaneous buildup of three samples (Figure S6), and a simultaneous microwave frequency/power sweep of three samples (Figure S9).


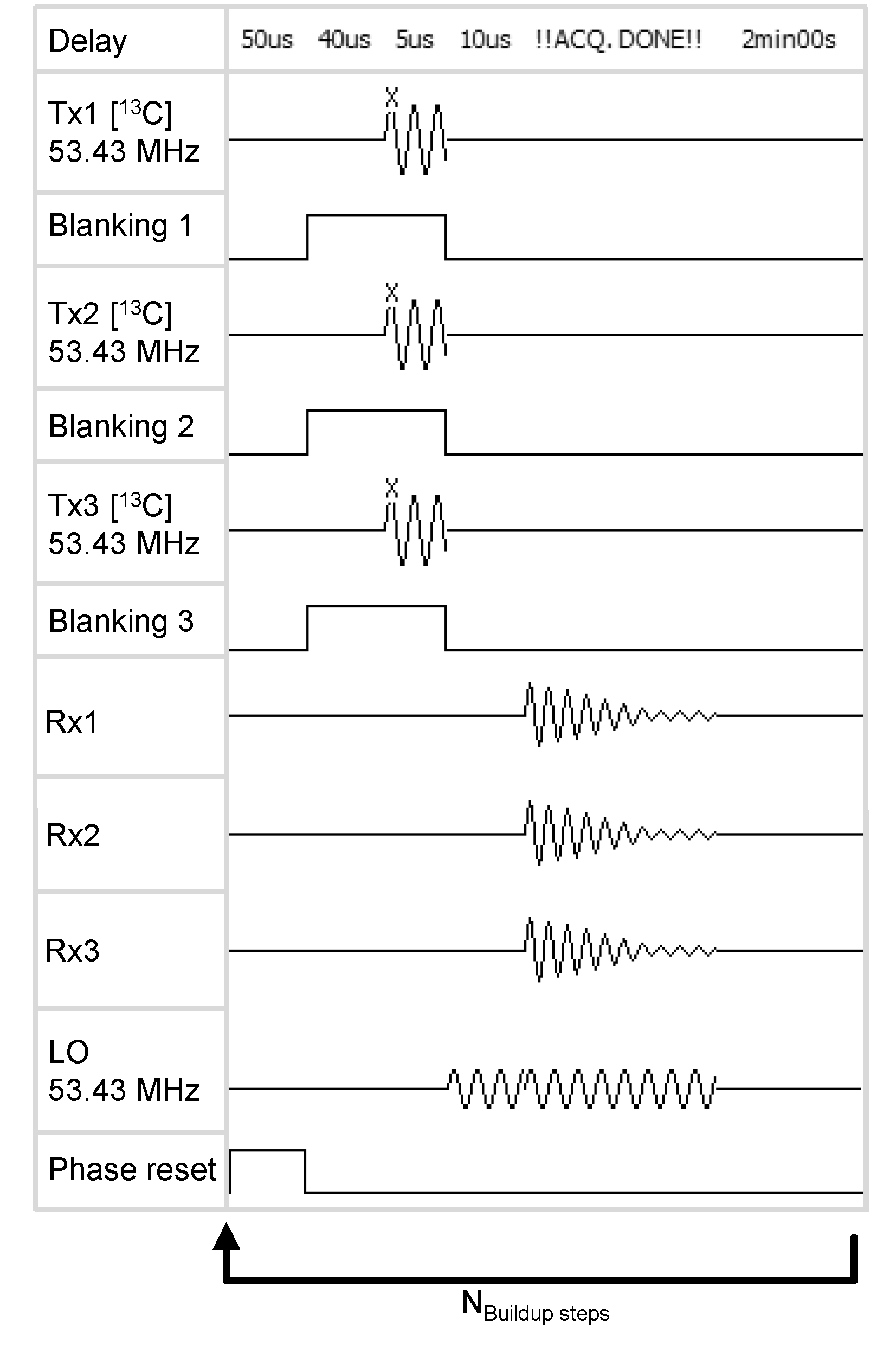


**Figure S4.** Sequence diagram for homonuclear buildup experiments. The three channels are excited (synchronous transmission) and read (synchronous reception) at the same time. A single local oscillator can be used to demodulate the signal read on the three channels at once because the frequency across the channel does not change.


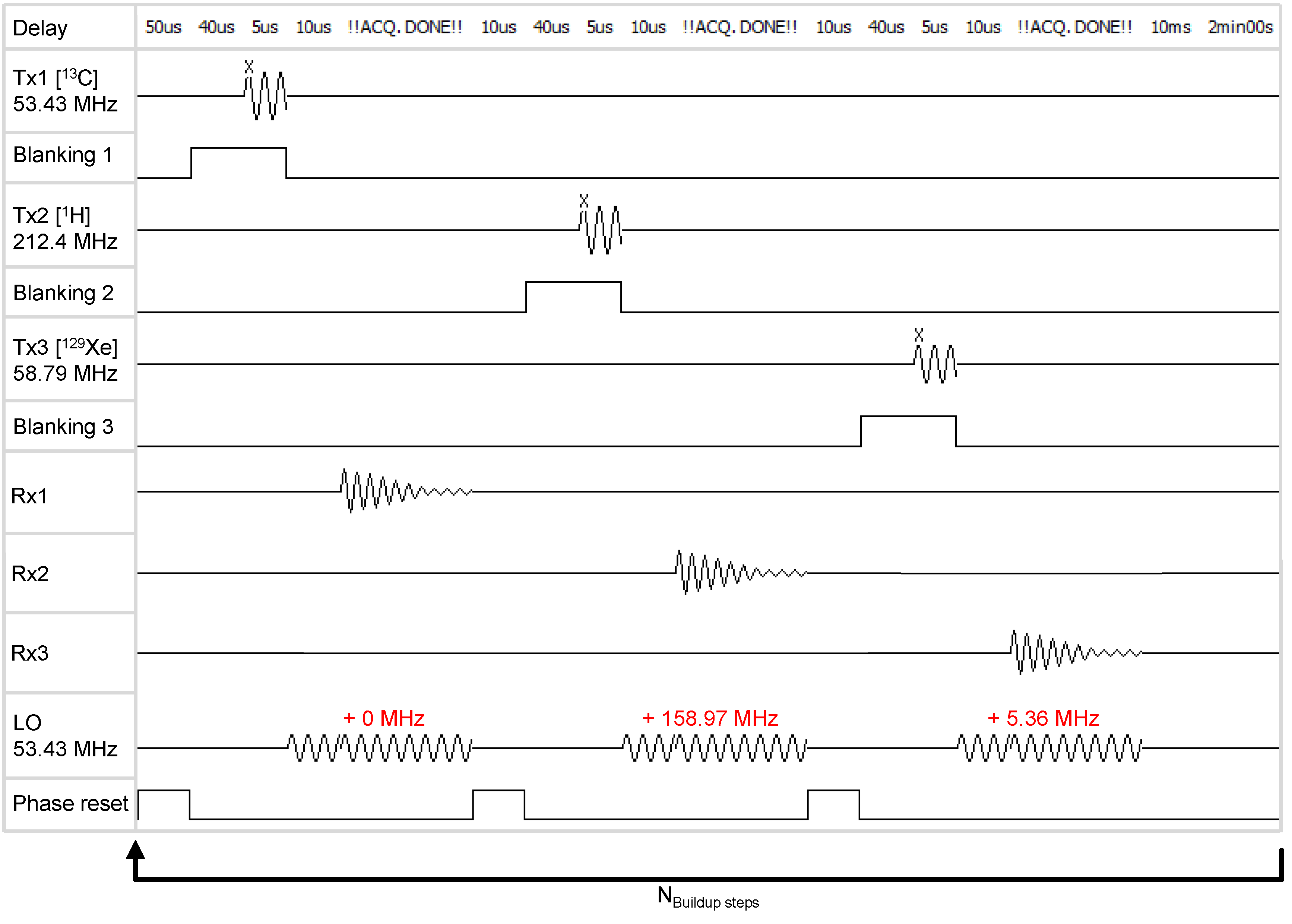


**Figure S5.** Sequence diagram for heteronuclear buildup experiments. The three channels are excited (asynchronous transmission) and read (synchronous reception) in sequence, with a delay <1 ms across the channels. A single local oscillator cannot be used to demodulate the signal read on the three channels at once because the demodulation frequency across the channels changes.


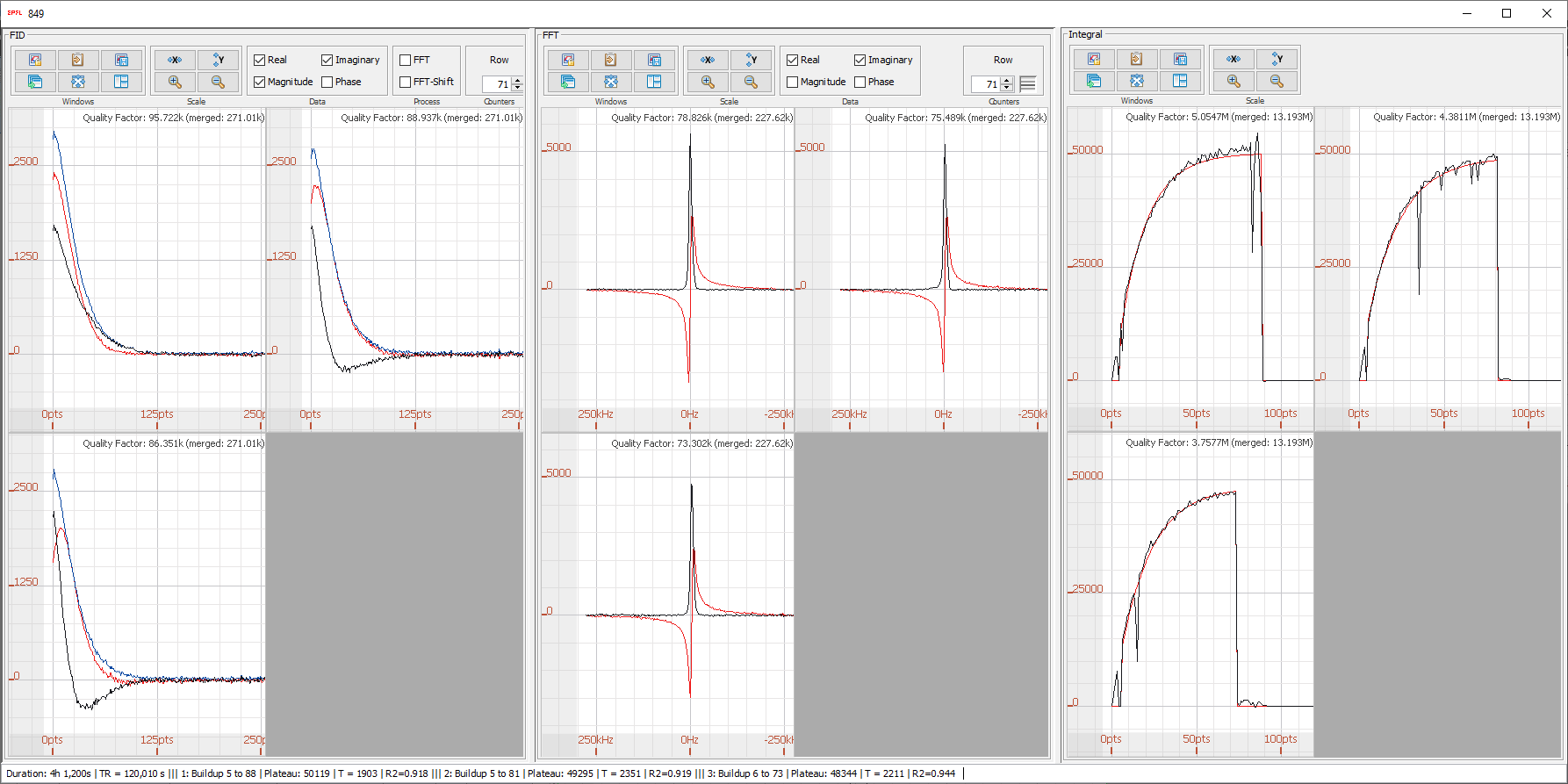


**Figure S6.** Plugin for simultaneous online data processing and visualization of the polarization buildup on the three channels. The first panel shows the three FIDs relative at a user-selected time point, the second panel the three spectra at a user-selected time point, and the third panel the time course evolution of the signal intensity from each channel.


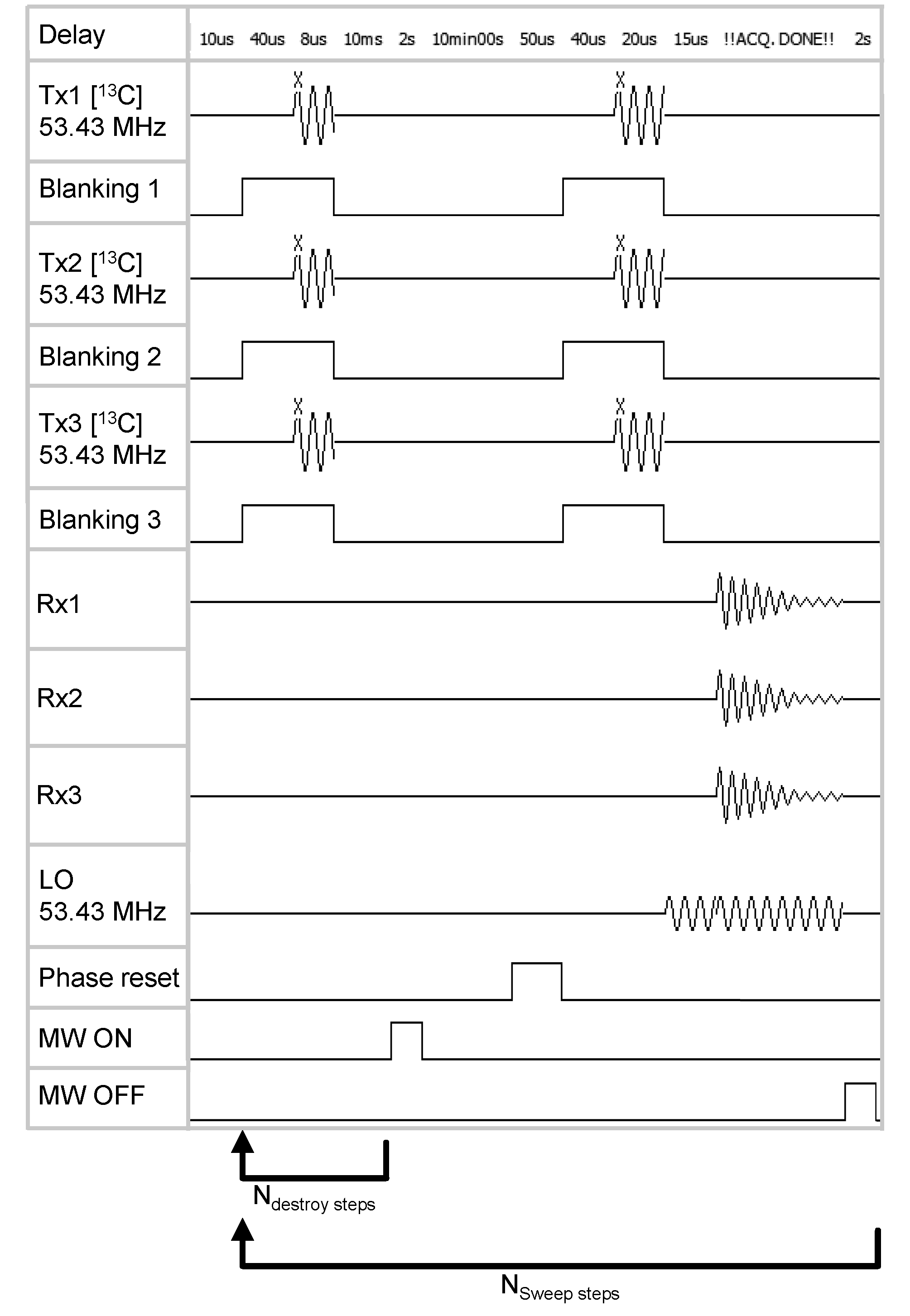


**Figure S7.** Sequence diagram for homonuclear microwave frequency/power sweep experiments. For each frequency/power step, firstly, the residual magnetization is destroyed on the three channels at the same time. Then, the three channels are excited (synchronous transmission) and read (synchronous reception) at the same time. A single local oscillator can be used to demodulate the signal read on the three channels at once because the frequency across the channel does not change.


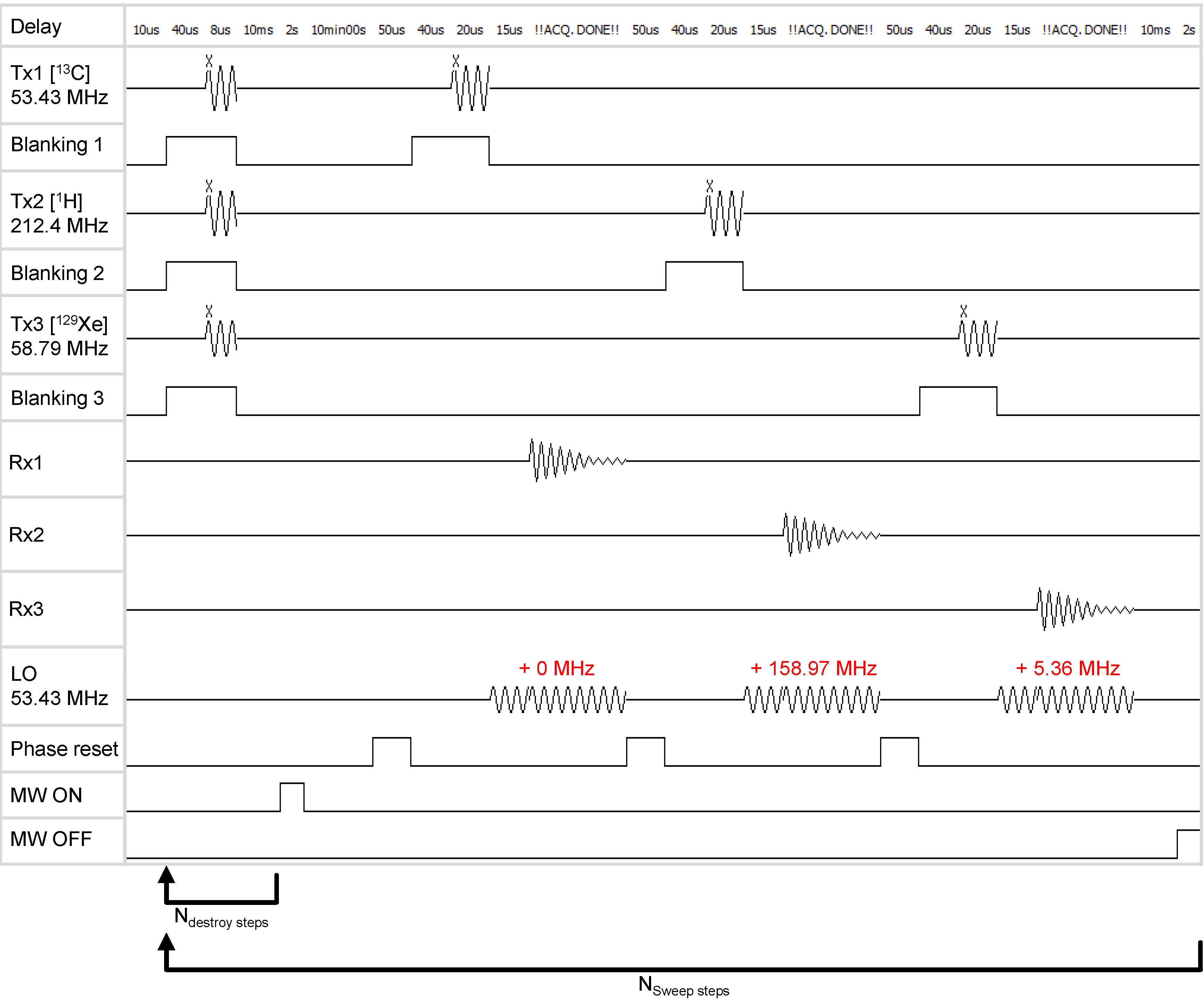


**Figure S8.** Sequence diagram for heteronuclear microwave frequency/power sweep experiments. For each frequency/power step, firstly, the residual magnetization is destroyed on the three channels at the same time. Then, the three channels are excited (asynchronous transmission) and read (synchronous reception) in sequence, with a delay <1 ms across the channels. A single local oscillator cannot be used to demodulate the signal read on the three channels at once because the demodulation frequency across the channels changes.


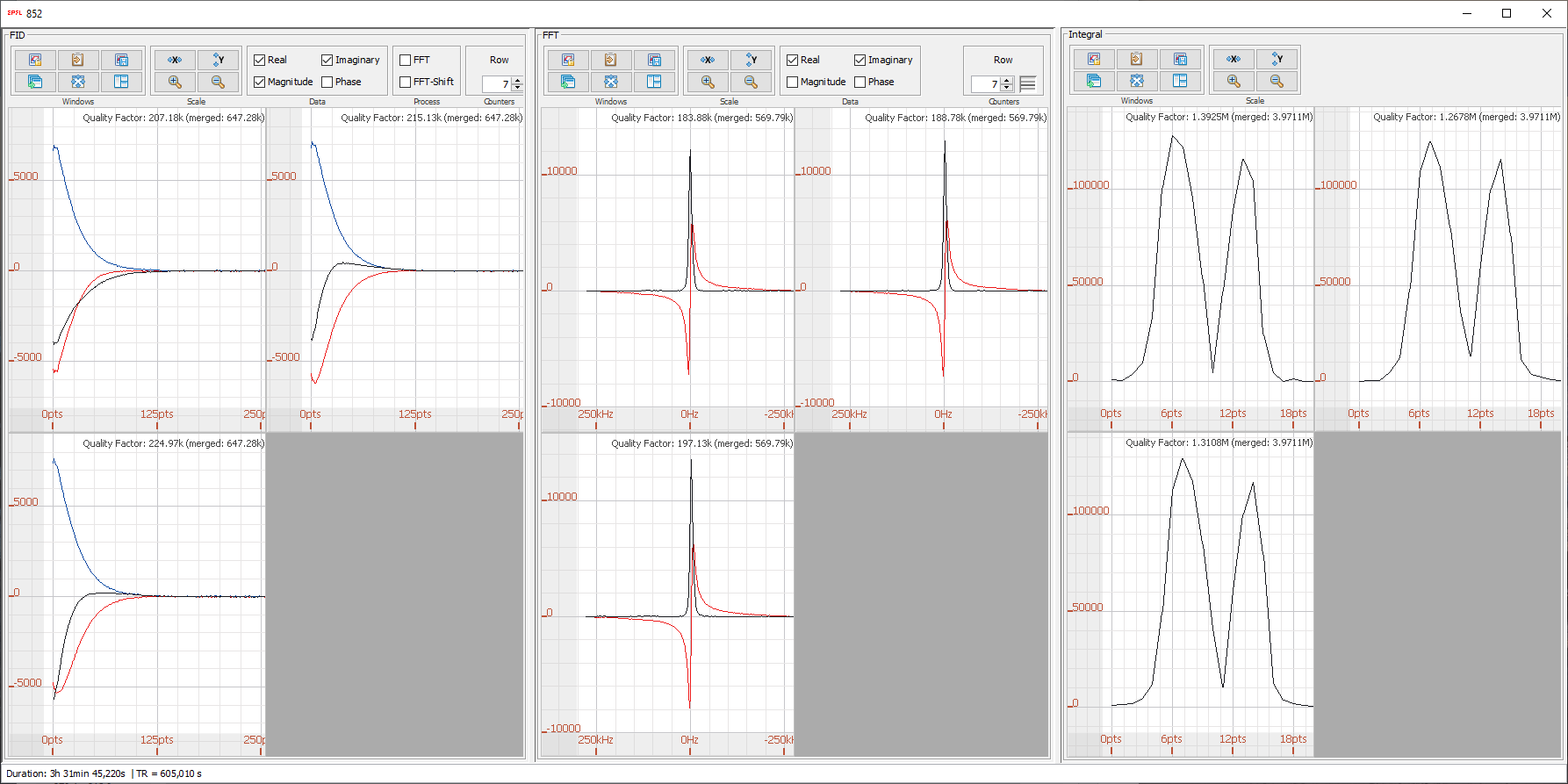


**Figure S9.** Plugin for simultaneous online data processing and visualization of the microwave frequency/power sweep on the three channels. The first panel shows the three FIDs at a user-selected frequency/power step, the second panel the three spectra at a user-selected frequency/power step, and the third panel the signal intensity as a function of the microwave frequency/power from each channel.

**3. Dissolution head description**

The dissolution head (Figure S10) heats up the dissolution buffer and releases it inside the CFP at high pressure. It is mounted above the polarizer on a double-swivel arm (Bosch Rexroth, Lohr am Main, Germany) to comfortably reach each slot of the multi-sample DNP probe.

The dissolution happens as follows. Firstly, 5.5 mL of buffer are loaded inside the boiler (E) through the loading valve (D), which is then closed. The boiler is pressurized via a set of check valves (A) using He gas released by the low-pressure He valve (K) at 4 bar pressure set using the pressure regulator (C). The 220 W heater band (Industrial Molding Supplies P/N 150975, Chagrin Falls, OH, USA) heats the boiler until the pressure transducer (I, Swagelok P/N PTI-S-AG16-32AS, Cleveland, OH, USA) reads 12.0 bar and stabilizes it within 0.5 bar of the target value, corresponding to a buffer temperature of about 180 °C. A 0.8 mm graphite thermal interface sheet ensures thermal contact between the boiler and the band heater.

In the meanwhile, the 100 W valve heater, a 10 Ω resistor (F, Ohmite P/N TGHDX10R0JE, Warrenville, IL, USA) stabilizes the temperature of both the quick-connect stem (H, Modified Swagelok P/N SS-QM2-2PM) and bottom dissolution pneumatic valve (G, Swagelok P/N 6LVV-DPVS6M-C) to 100 °C measured by a PT100 thermometer.

The user lifts the CFP by 10 cm to take the CFP vial away from liquid He, then docks the CFP to the quick-connect stem (H) and presses the dissolution button (B). An automated LabVIEW computer-controlled sequence opens the bottom dissolution valve to release the buffer into the CFP to melt the sample. Simultaneously, the top dissolution pneumatic valve (J) opens and He gas at 10 bar is blown through the CFP to eject and transfer the sample to the separator/infusion pump in the MRI scanner. After 5000 ms both valves close, the MRI acquisition is automatically triggered, and the user can disconnect the CFP and remove it from the polarizer. Dissolutions to the benchtop spectrometer require 2500 ms pushing time at 8 bar.

Following the dissolution, the boiler and bottom valve are cooled down and dried using compressed air flushed from (A) to (H). Fiberglass insulation (not shown) is wrapped around all hot components to prevent contact burns.

**Figure S10** : Dissolution head. (**A**) Low-pressure helium and air inlet, (**B**) Dissolution button, (**C**) Pressure regulator for low helium gas, (**D**) Loading valve for dissolution solvent, (**E**) Water boiler and 220 W band heater, (**F**) 100 W valve heater, (**G**) Bottom dissolution pneumatic valve, (**H**) Quick-connect stem, (**I**) Pressure transducer (behind), (**J**) Top dissolution pneumatic valve, (**K**) Low-pressure helium pneumatic valve, (**L**) Swivel and ball screw.

**4. Evaluation of cross talk between the NMR channels during homonuclear experiments**

In Figure S11 we report individual NMR spectra acquired during the dissolution DNP experiment with three PA-samples presented in Section 3.1 and Figure 5 of the main manuscript. An identical vertical scale was applied to all plots. All integrals were normalized to the largest integral value. 5 µl PA-samples were loaded into each slot of the multi-sample DNP probe, then hyperpolarized at 139.87 GHz and 63 mW power. Every 120 s, the NMR signal from the three samples was simultaneously acquired with a 5° hard pulse sent on each sample, and the NMR signal parallelly detected (described in Figure S4). The first row (A) displays the spectra acquired at 140 min after the start of the experiment. The NMR signal intensity is similar within 7% discrepancy across the slots. After dissolving the sample in slot 3 (B, at 156 min), a signal equivalent to 3.6% of the signal prior to dissolution is observed in that slot. This residual NMR signal is likely detected from the samples in slots 1 and 2. After the second dissolution (C, at 170 min), a similar residual signal is observed in slots 2 and 3. No NMR signal is observed following the dissolution of all samples (D, 180 min timepoint).


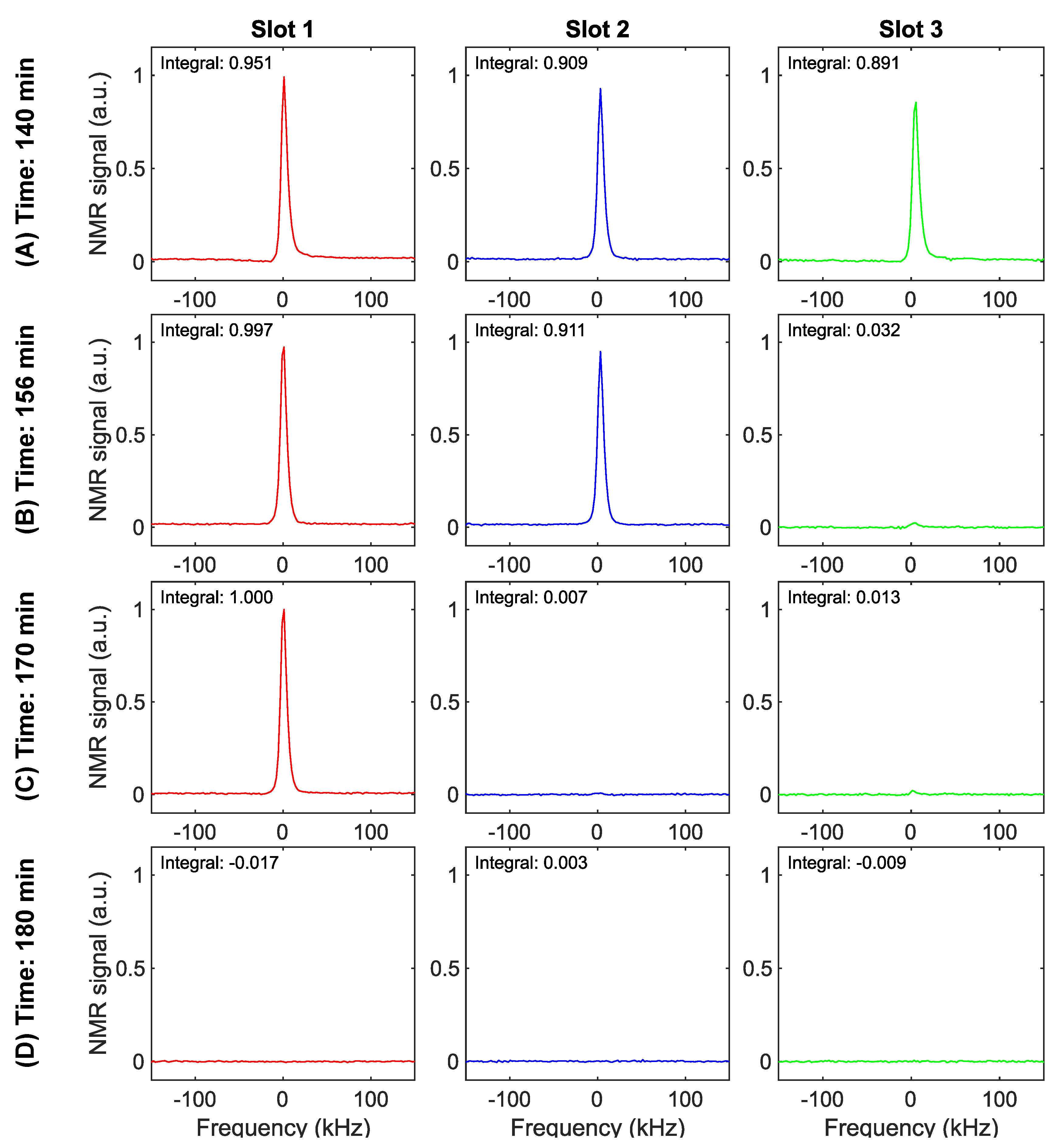


**Figure S11.** Investigation of cross talk between the three slots during a homonuclear experiment. The first row (A) displays the spectra acquired at 140 min after the start of the experiment. The NMR signal intensity is similar within 7% discrepancy across the slots. After dissolving the sample in slot 3 (B, at 156 min), a signal equivalent to 3.6% of the signal prior to dissolution is observed in that slot. This residual NMR signal is likely detected from the samples in slots 1 and 2. After the second dissolution (C, at 170 min), a similar residual signal is observed in slots 2 and 3. No NMR signal is observed following the dissolution of all samples (D, 180 min timepoint).

**5. PA-sample and Lac-sample microwave frequency sweep**

In Figure S12 we report the microwave frequency sweep of the Lac-sample and PA-sample measured simultaneously using the multi-sample DNP probe. Both samples have maximum enhancement at 139.86 GHz. The spectral breadth of the Lac-sample is narrower because of the presence of Gd^3+^ its formulation.


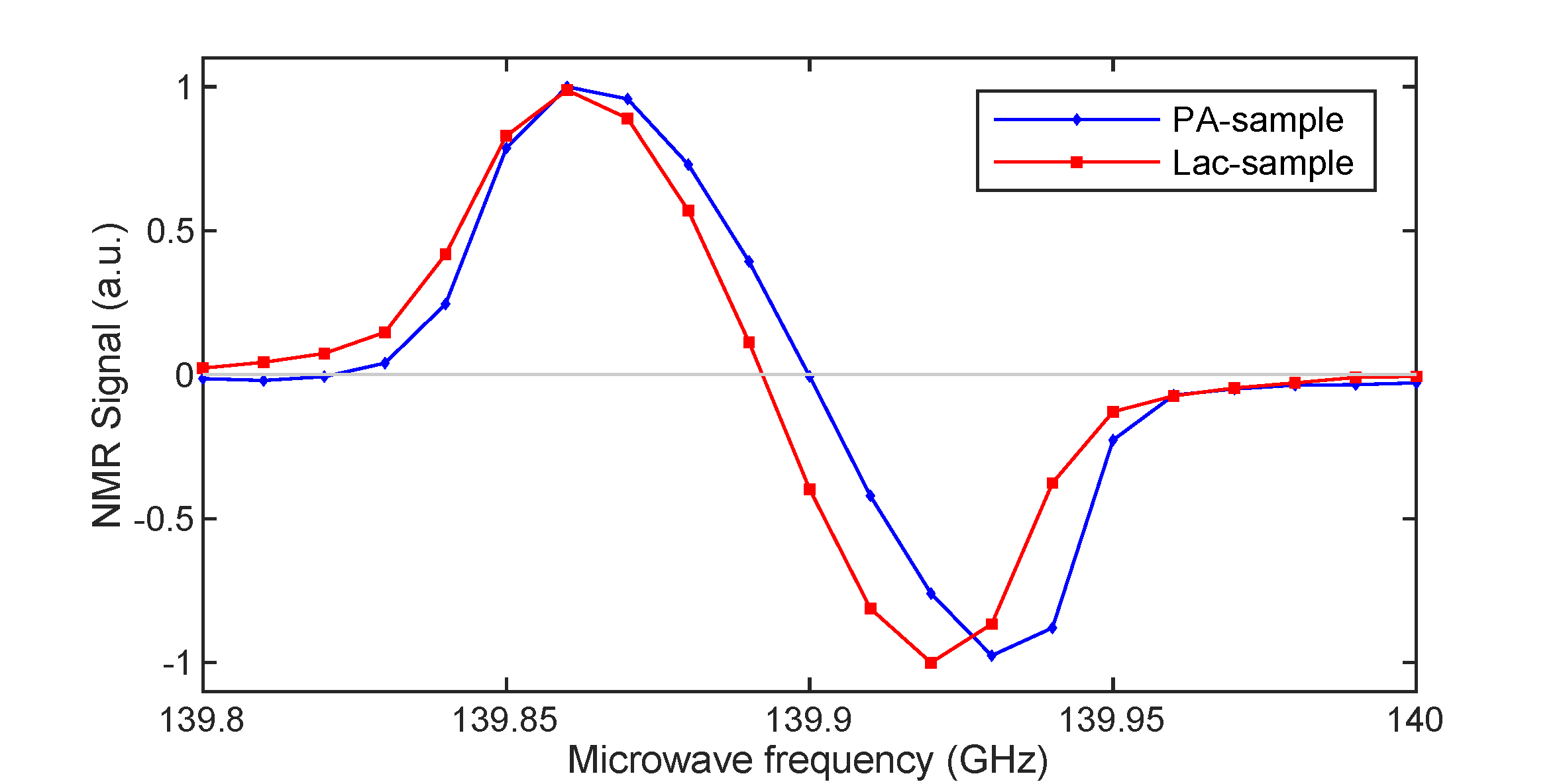


**Figure S12.** Simultaneous microwave frequency sweep of the Lac-sample and PA-sample from 139.8 GHz to 140.0 GHz.
